# Supplementary material for: The Erlang distribution approximates the age distribution of incidence of childhood and young adulthood cancers
Source: PeerJ. 2021 Aug 6;9:e11976. doi: 10.7717/peerj.11976 (PMC8351573; doi:10.7717/peerj.11976)
Supplement: Supplemental Information 1 [file peerj-09-11976-s001.pdf]

Supplemental Material for "The Erlang distribution approximates the age distribution of incidence of childhood and young adulthood cancers" by Aleksey V. Belikov, Alexey D. Vyatkin and Sergey V. Leonov

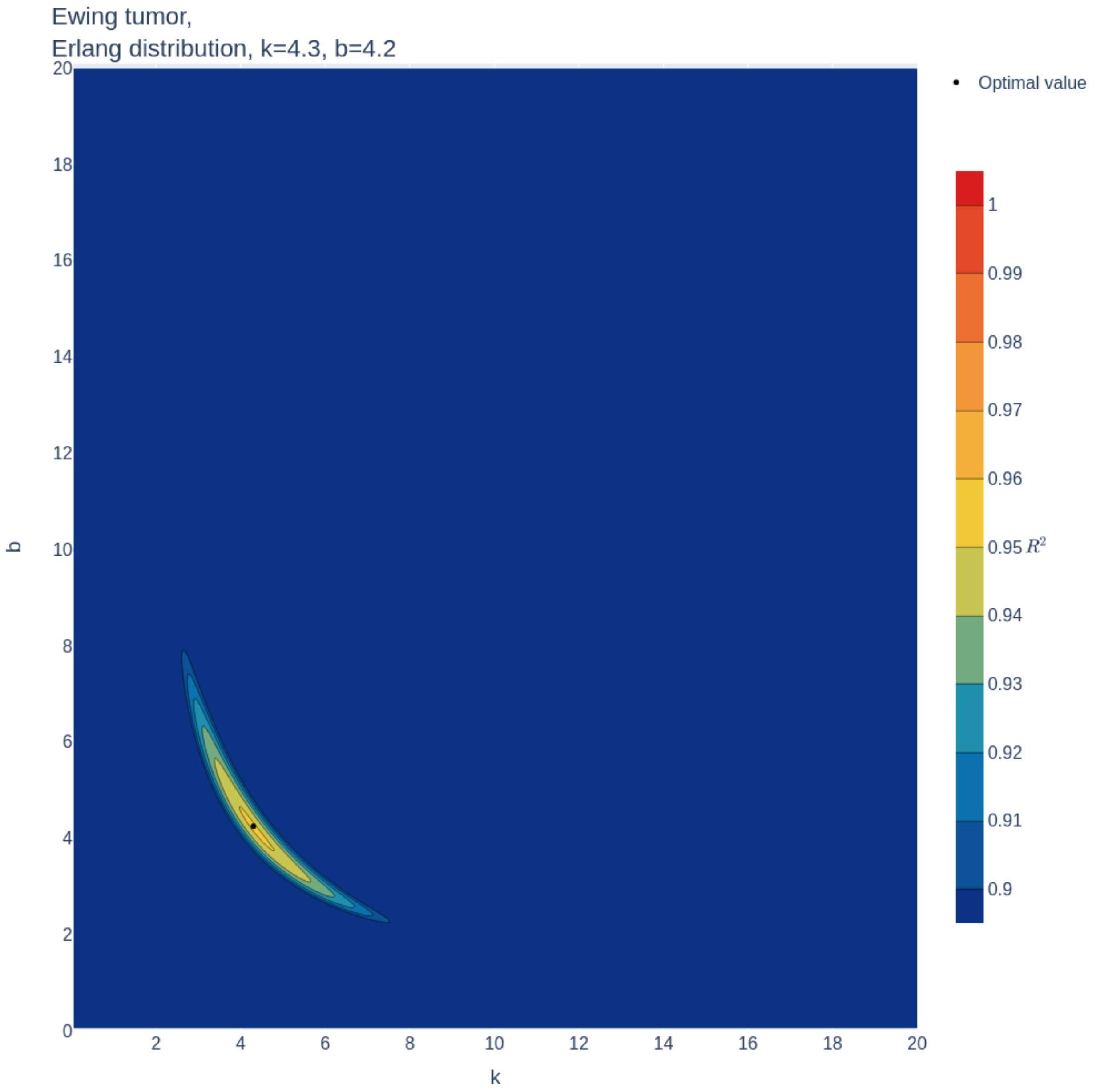

Web Figure 1. Goodness of fit of the gamma/Erlang distribution to the age distribution of incidence of Ewing tumor as a function of various parameter combinations.

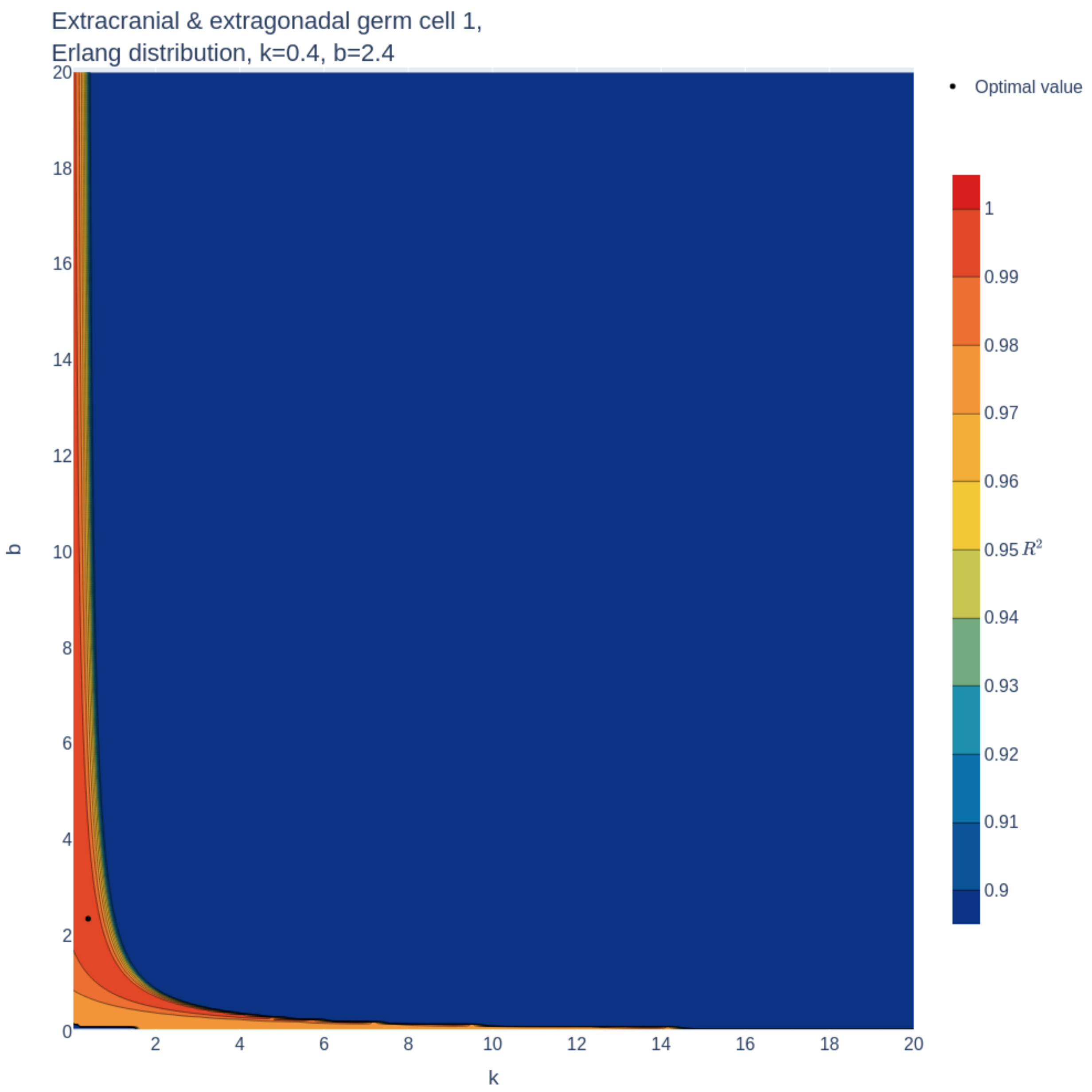

Web Figure 2. Goodness of fit of the gamma/Erlang distribution to the age distribution of incidence of extracranial and extragonadal germ cell tumors of childhood as a function of various parameter combinations.

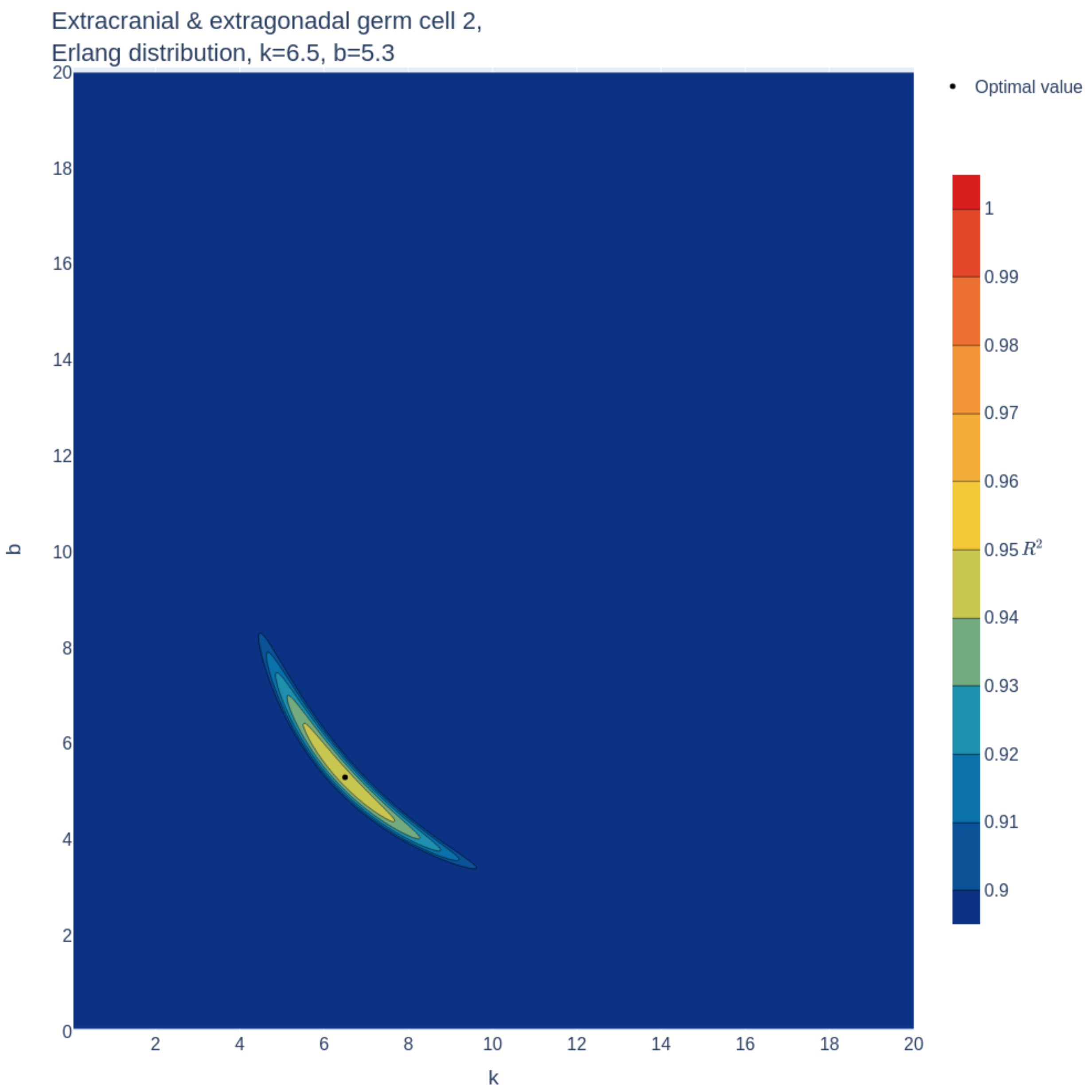

Web Figure 3. Goodness of fit of the gamma/Erlang distribution to the age distribution of incidence of extracranial and extragonadal germ cell tumors of young adulthood as a function of various parameter combinations.

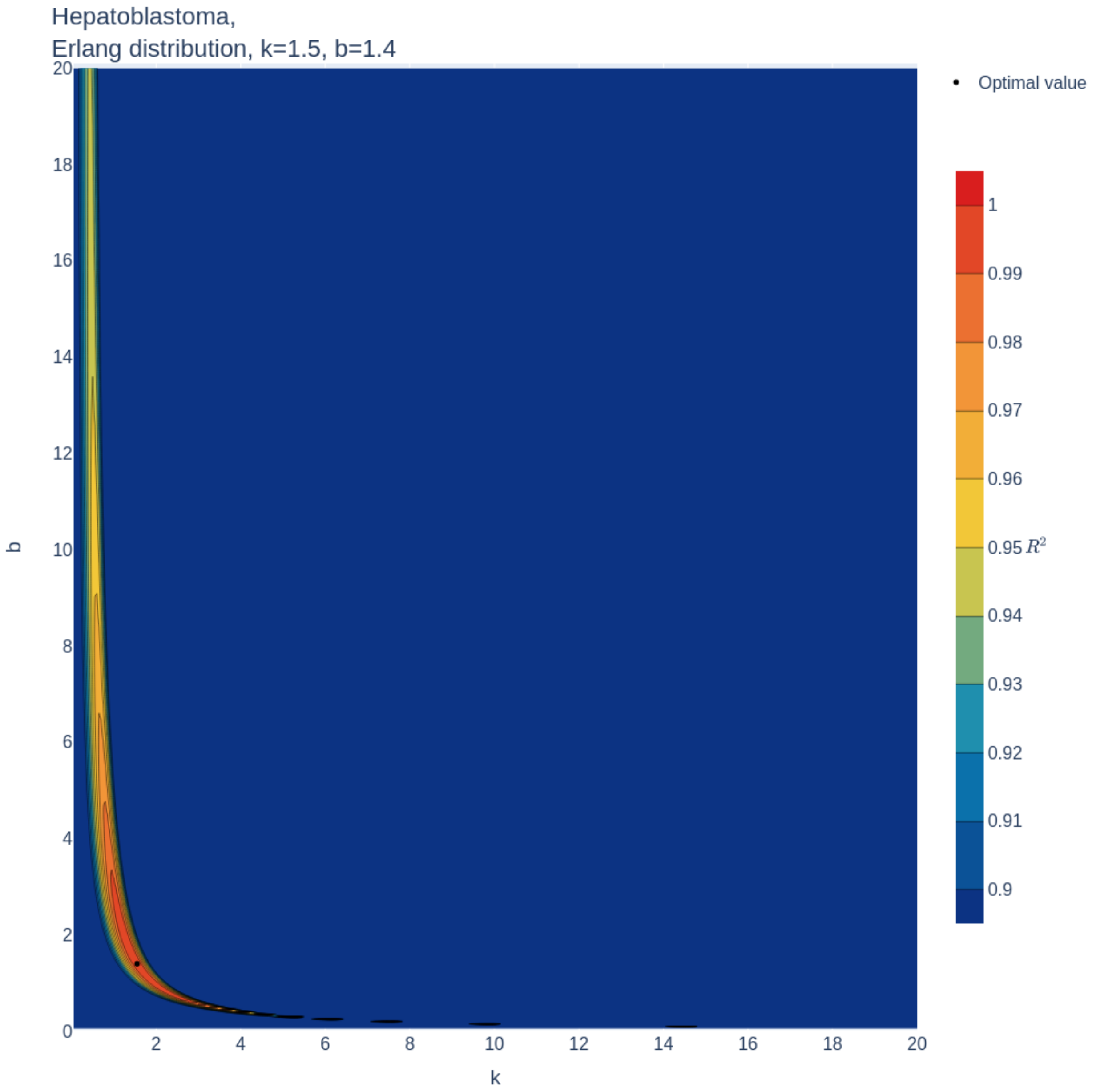

Web Figure 4. Goodness of fit of the gamma/Erlang distribution to the age distribution of incidence of hepatoblastoma as a function of various parameter combinations.

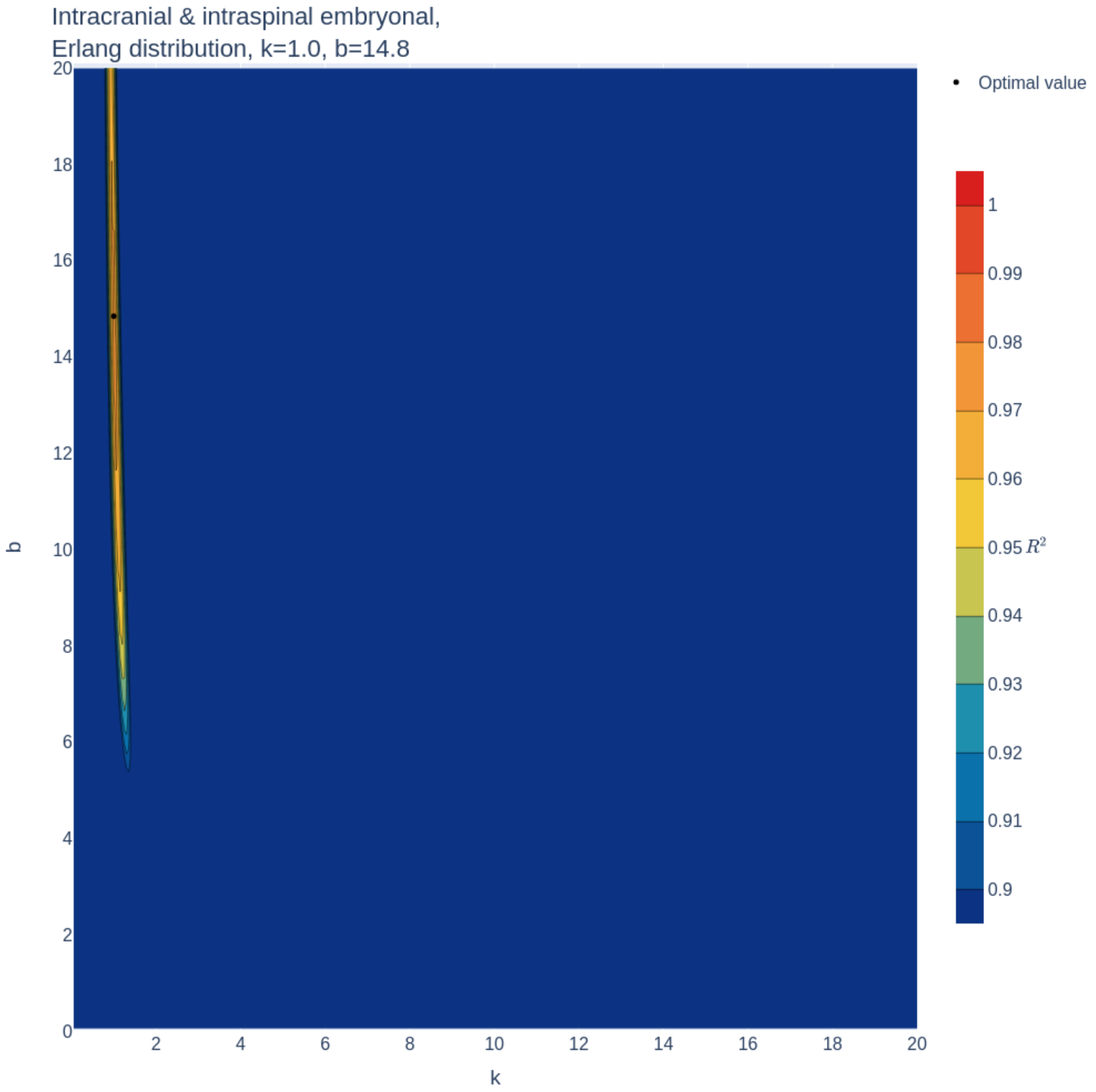

Web Figure 5. Goodness of fit of the gamma/Erlang distribution to the age distribution of incidence of intracranial and intraspinal embryonal tumors as a function of various parameter combinations.

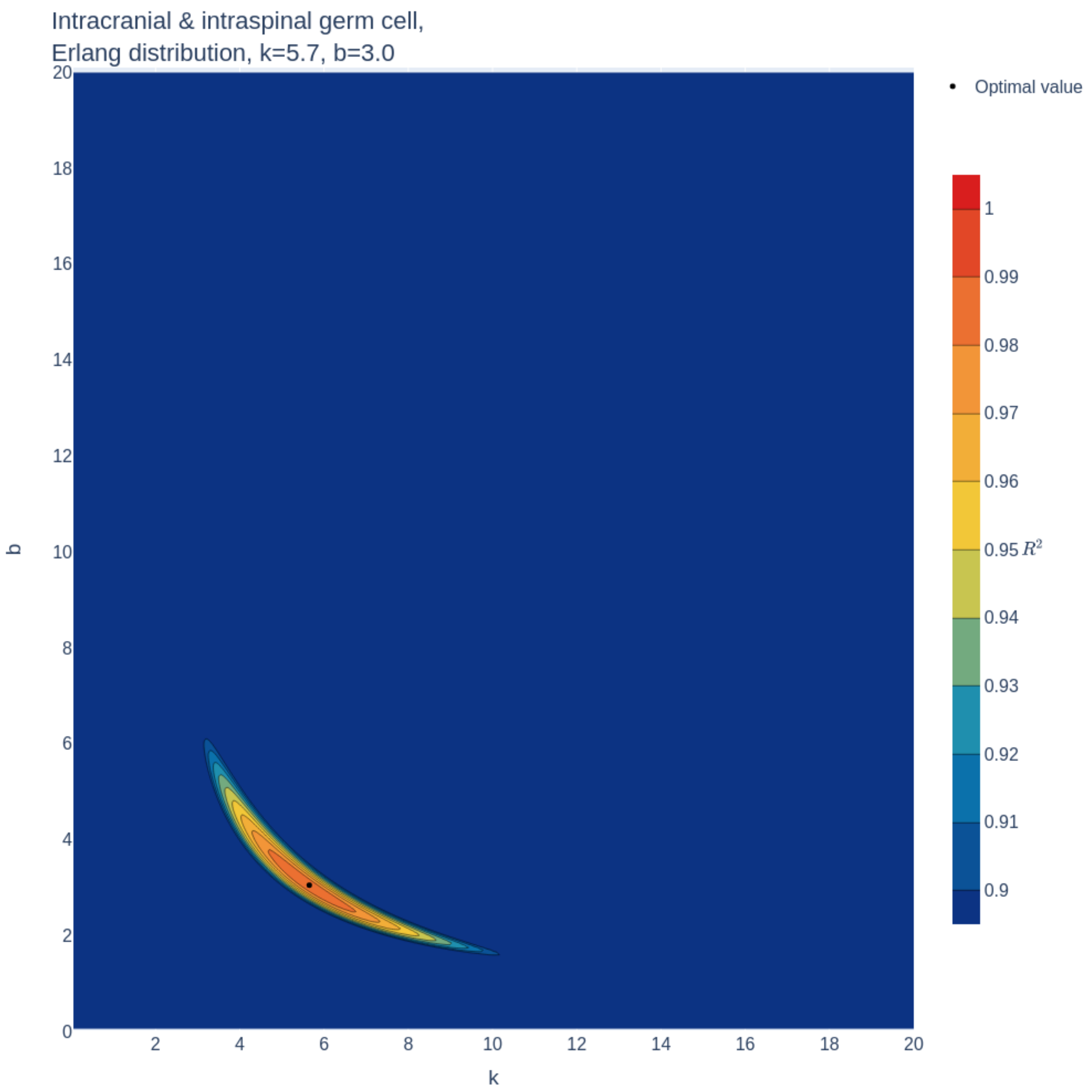

Web Figure 6. Goodness of fit of the gamma/Erlang distribution to the age distribution of incidence of intracranial and intraspinal germ cell tumors as a function of various parameter combinations.

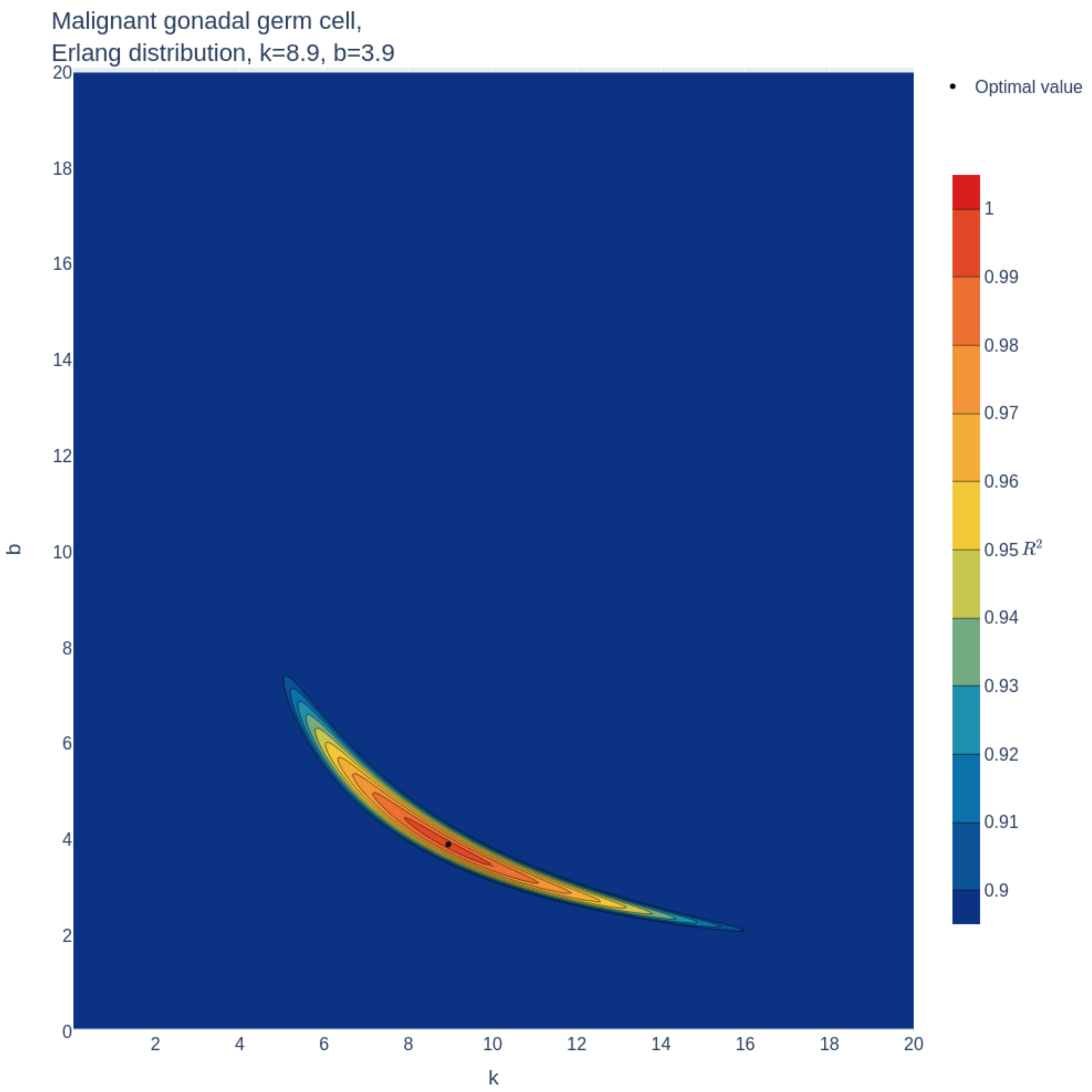

Web Figure 7. Goodness of fit of the gamma/Erlang distribution to the age distribution of incidence of malignant gonadal germ cell tumors as a function of various parameter combinations.

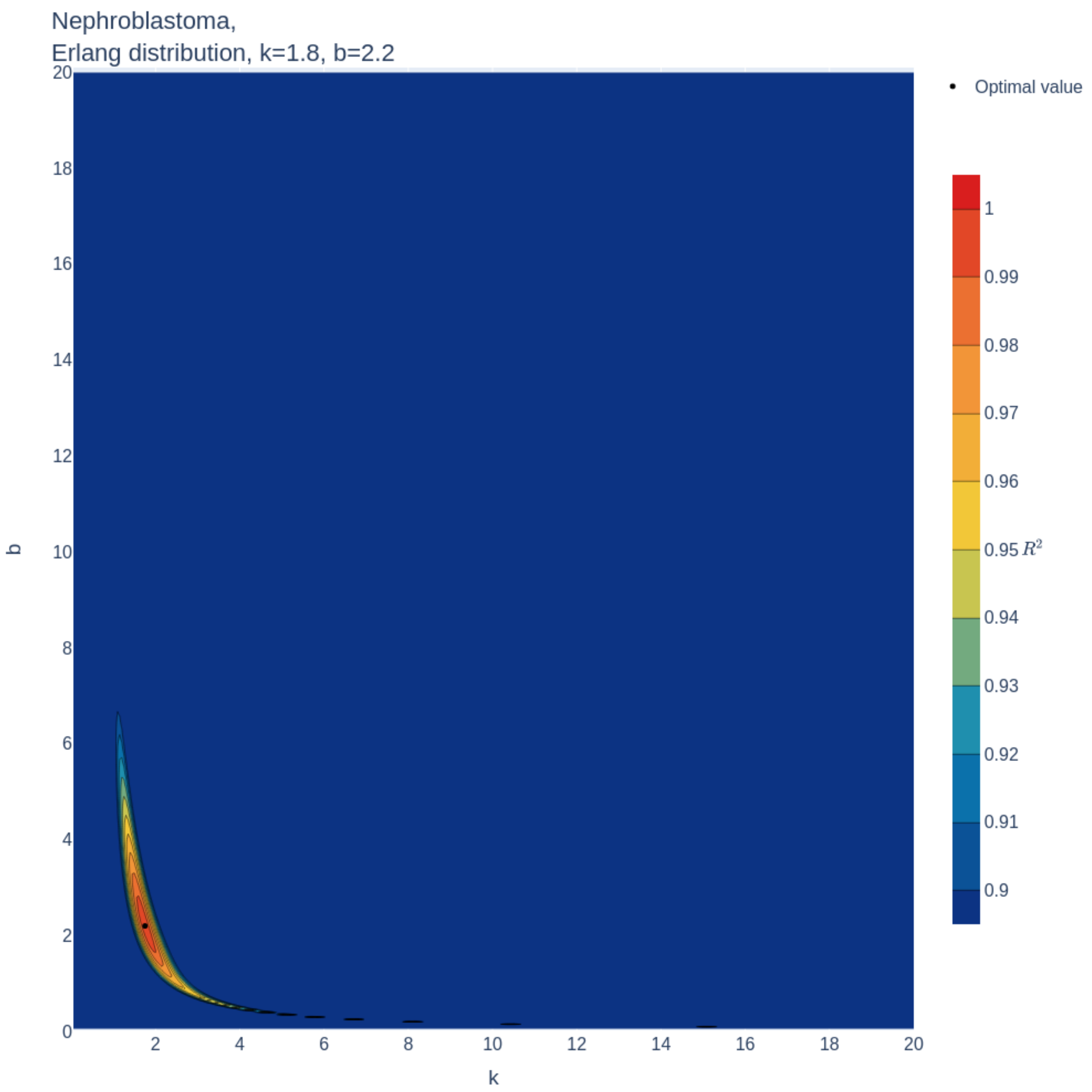

Web Figure 8. Goodness of fit of the gamma/Erlang distribution to the age distribution of incidence of nephroblastoma as a function of various parameter combinations.

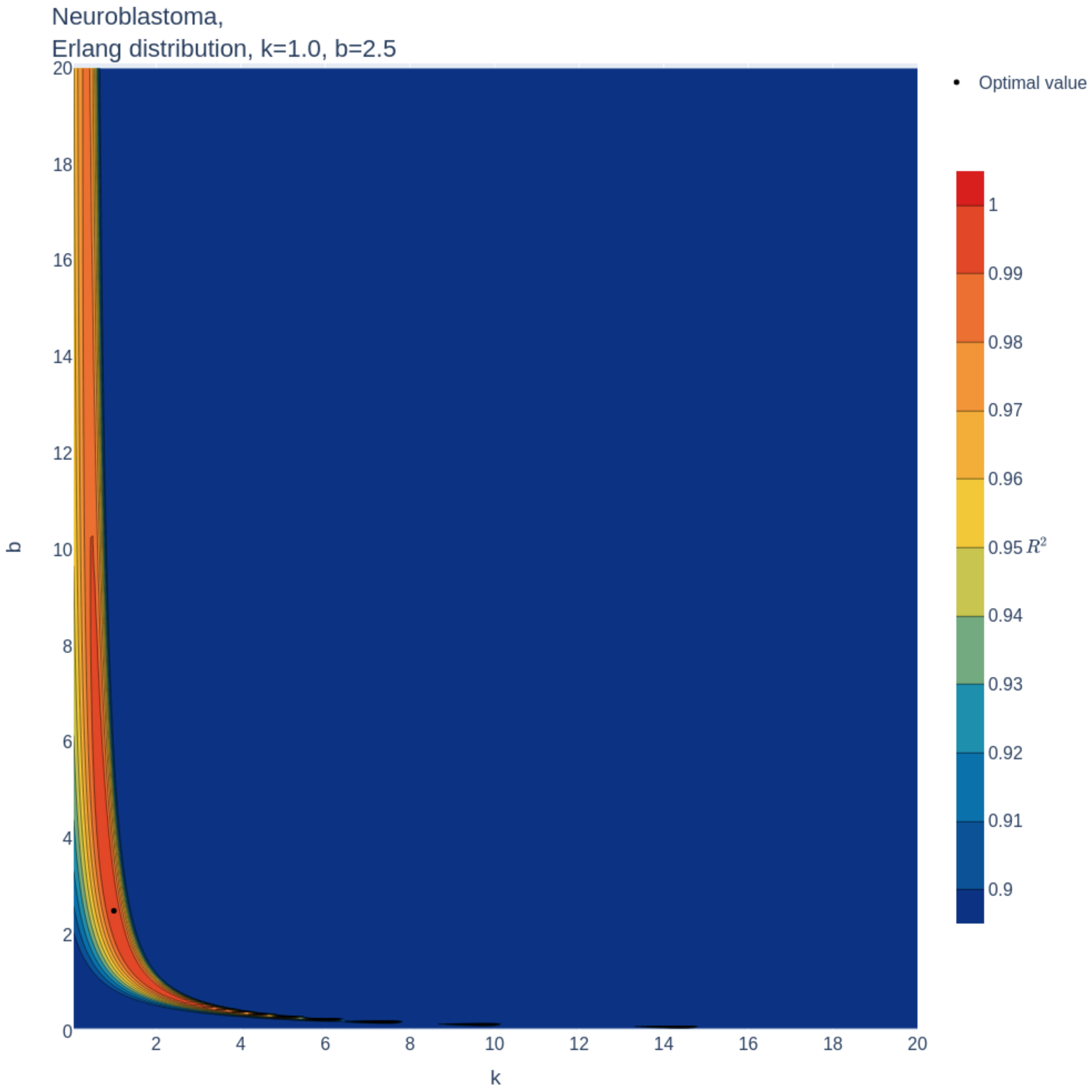

Web Figure 9. Goodness of fit of the gamma/Erlang distribution to the age distribution of incidence of neuroblastoma as a function of various parameter combinations.

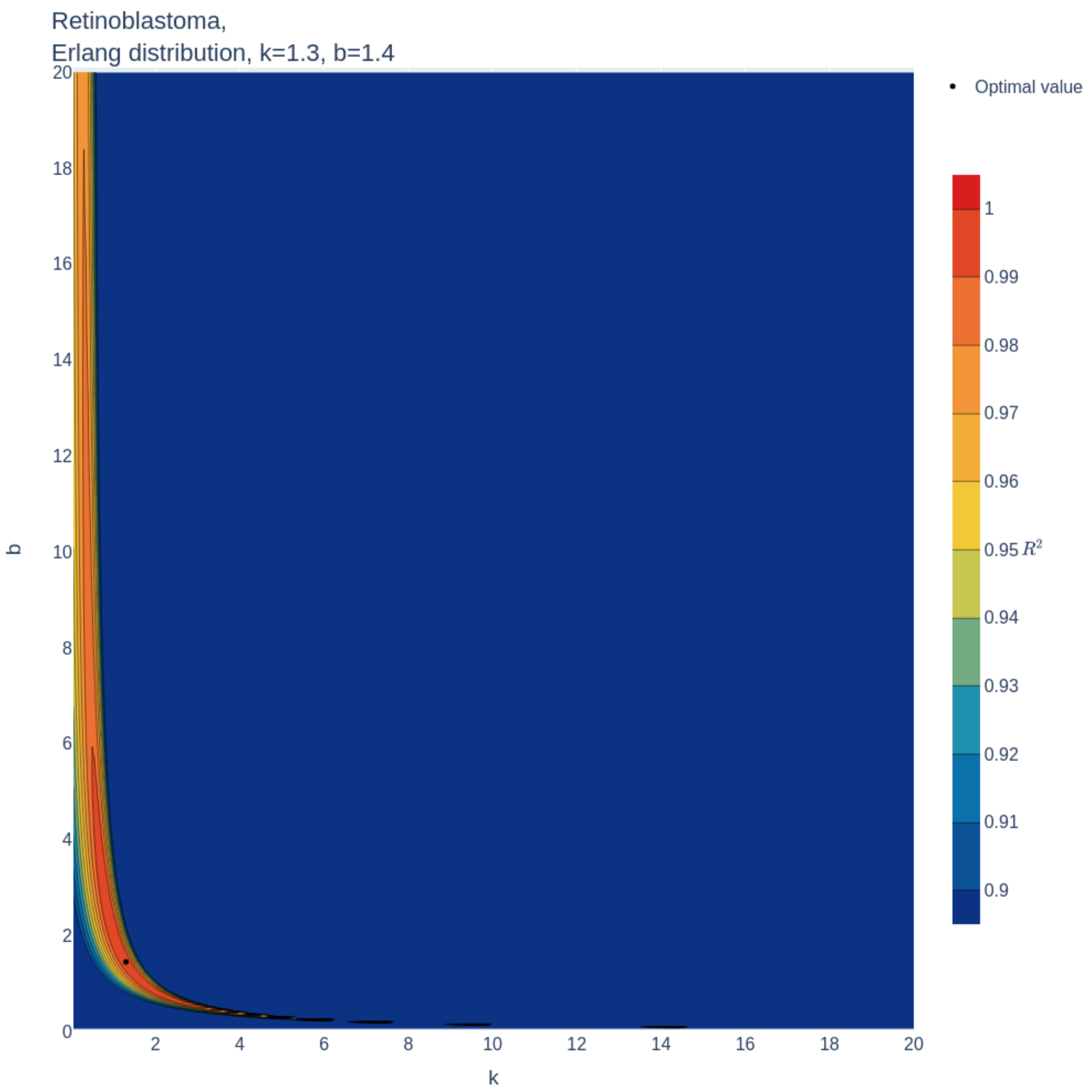

Web Figure 10. Goodness of fit of the gamma/Erlang distribution to the age distribution of incidence of retinoblastoma as a function of various parameter combinations.

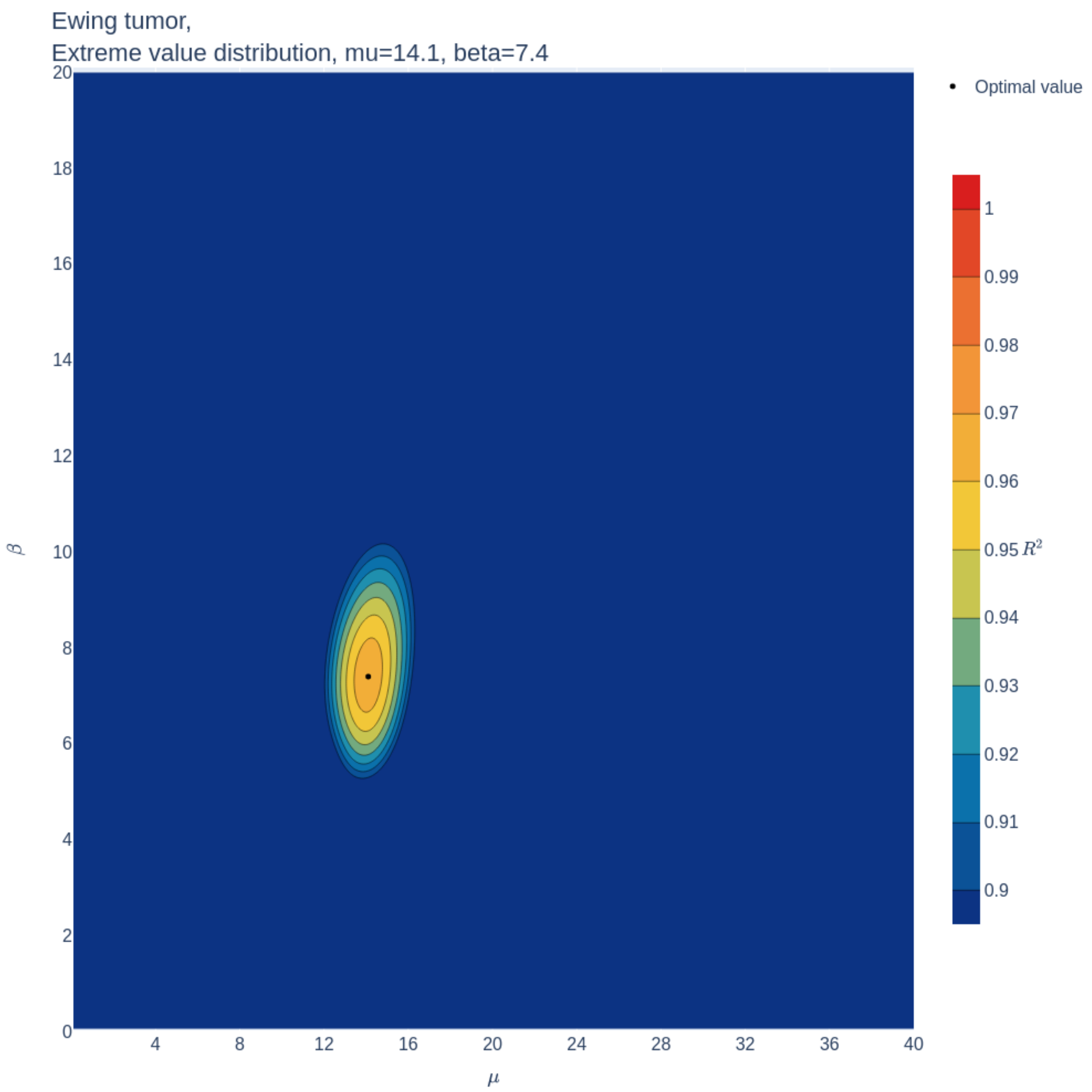

Web Figure 11. Goodness of fit of the extreme value distribution to the age distribution of incidence of Ewing tumor as a function of various parameter combinations.

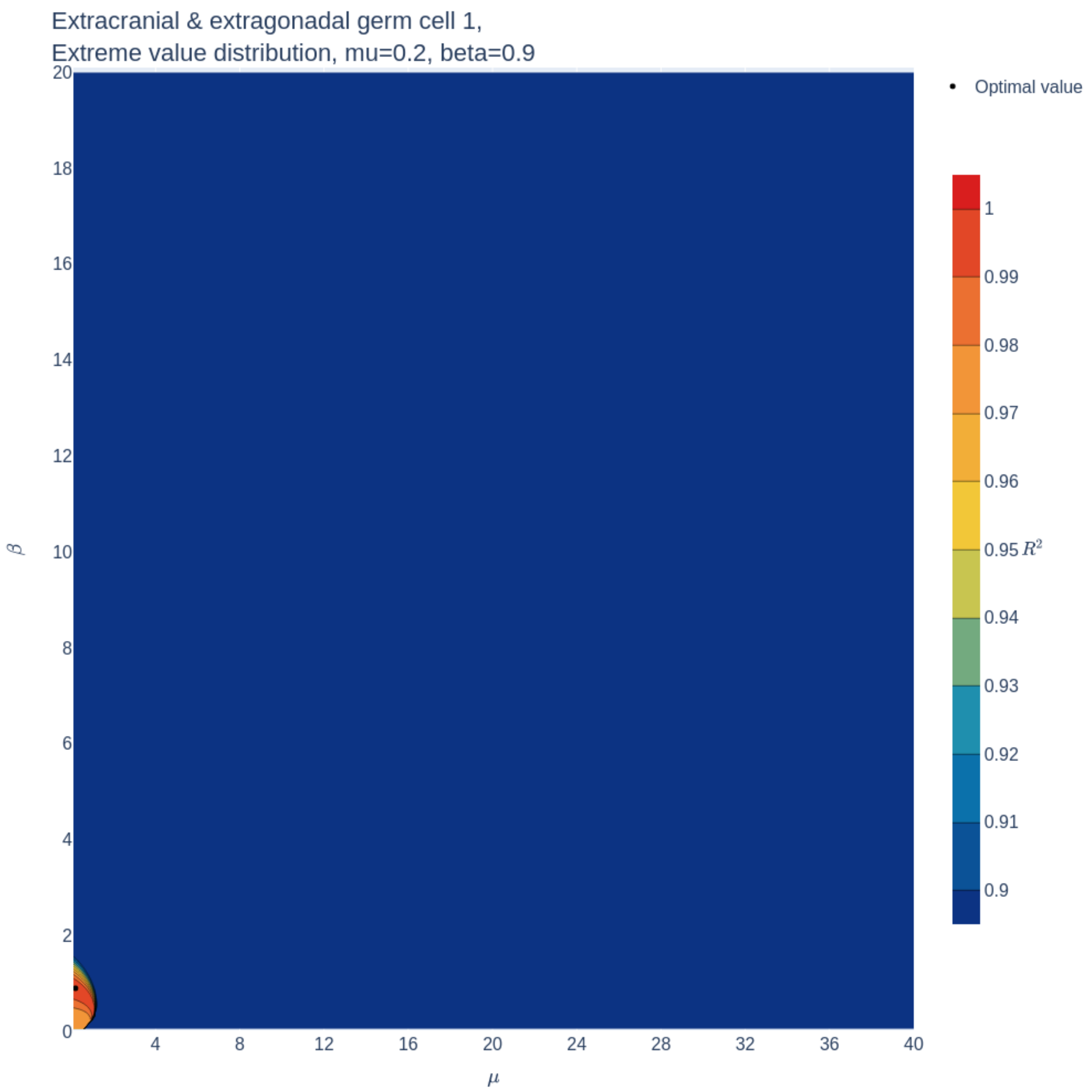

Web Figure 12. Goodness of fit of the extreme value distribution to the age distribution of incidence of extracranial and extragonadal germ cell tumors of childhood as a function of various parameter combinations.

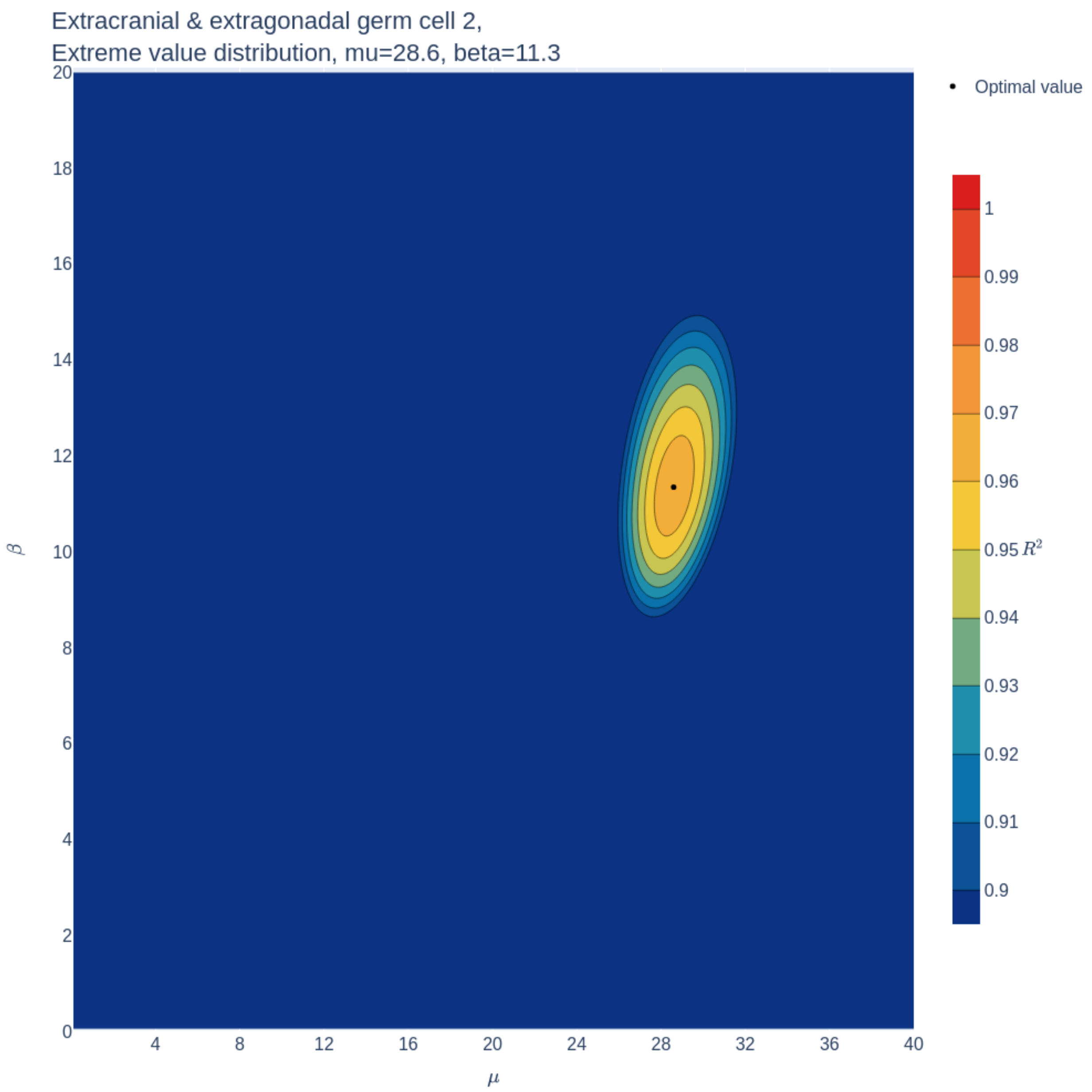

Web Figure 13. Goodness of fit of the extreme value distribution to the age distribution of incidence of extracranial and extragonadal germ cell tumors of young adulthood as a function of various parameter combinations.

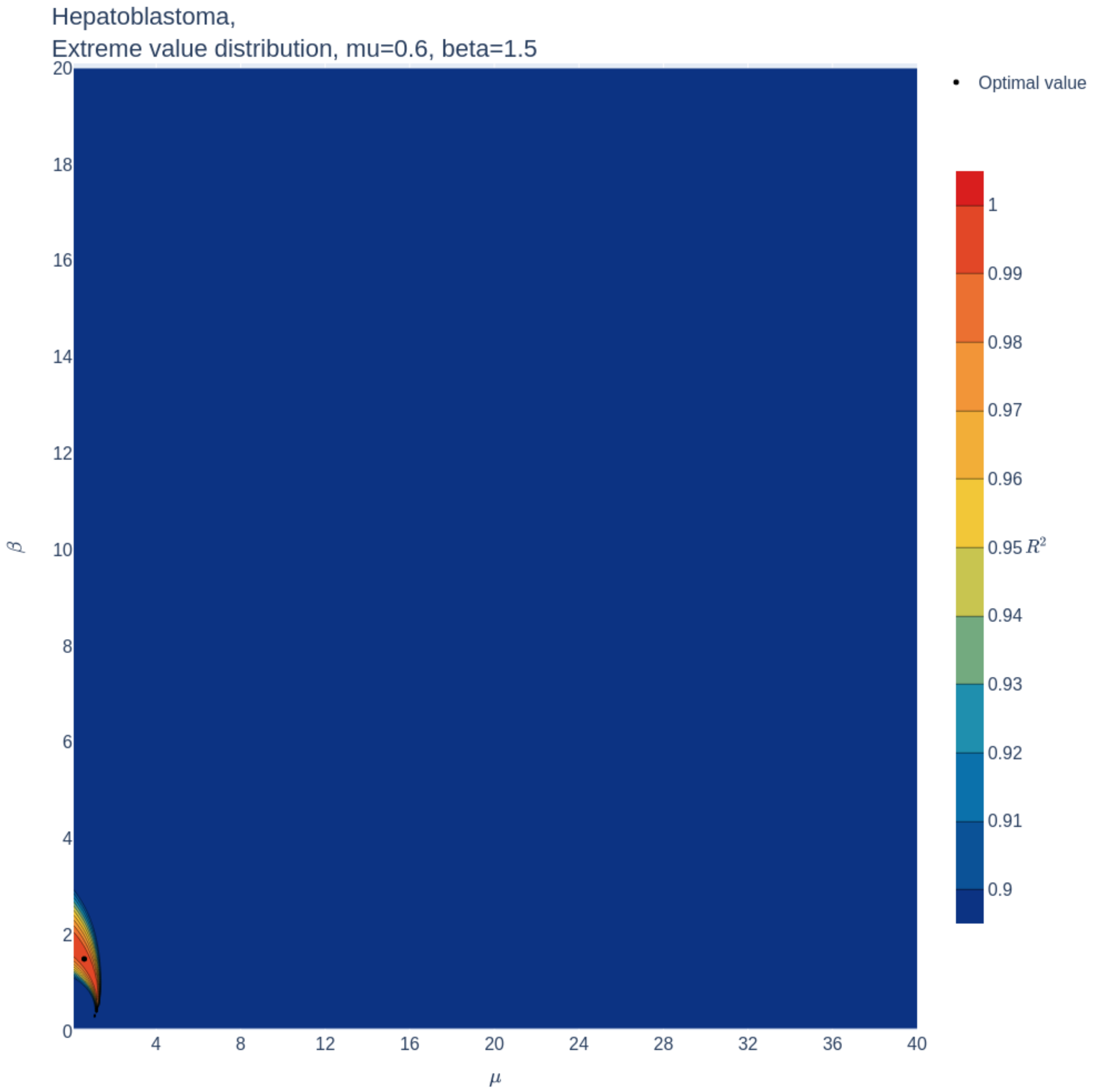

Web Figure 14. Goodness of fit of the extreme value distribution to the age distribution of incidence of hepatoblastoma as a function of various parameter combinations.

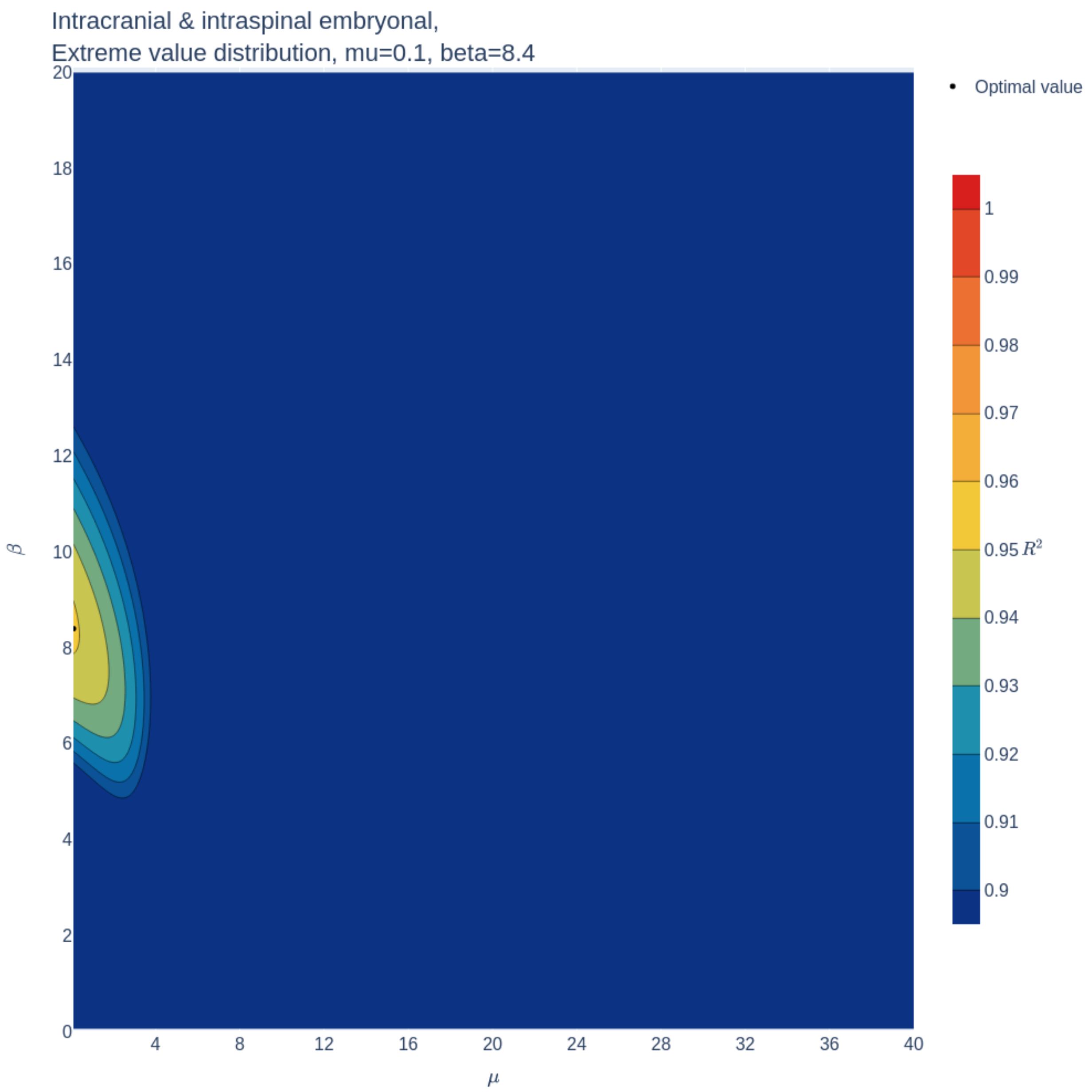

Web Figure 15. Goodness of fit of the extreme value distribution to the age distribution of incidence of intracranial and intraspinal embryonal tumors as a function of various parameter combinations.

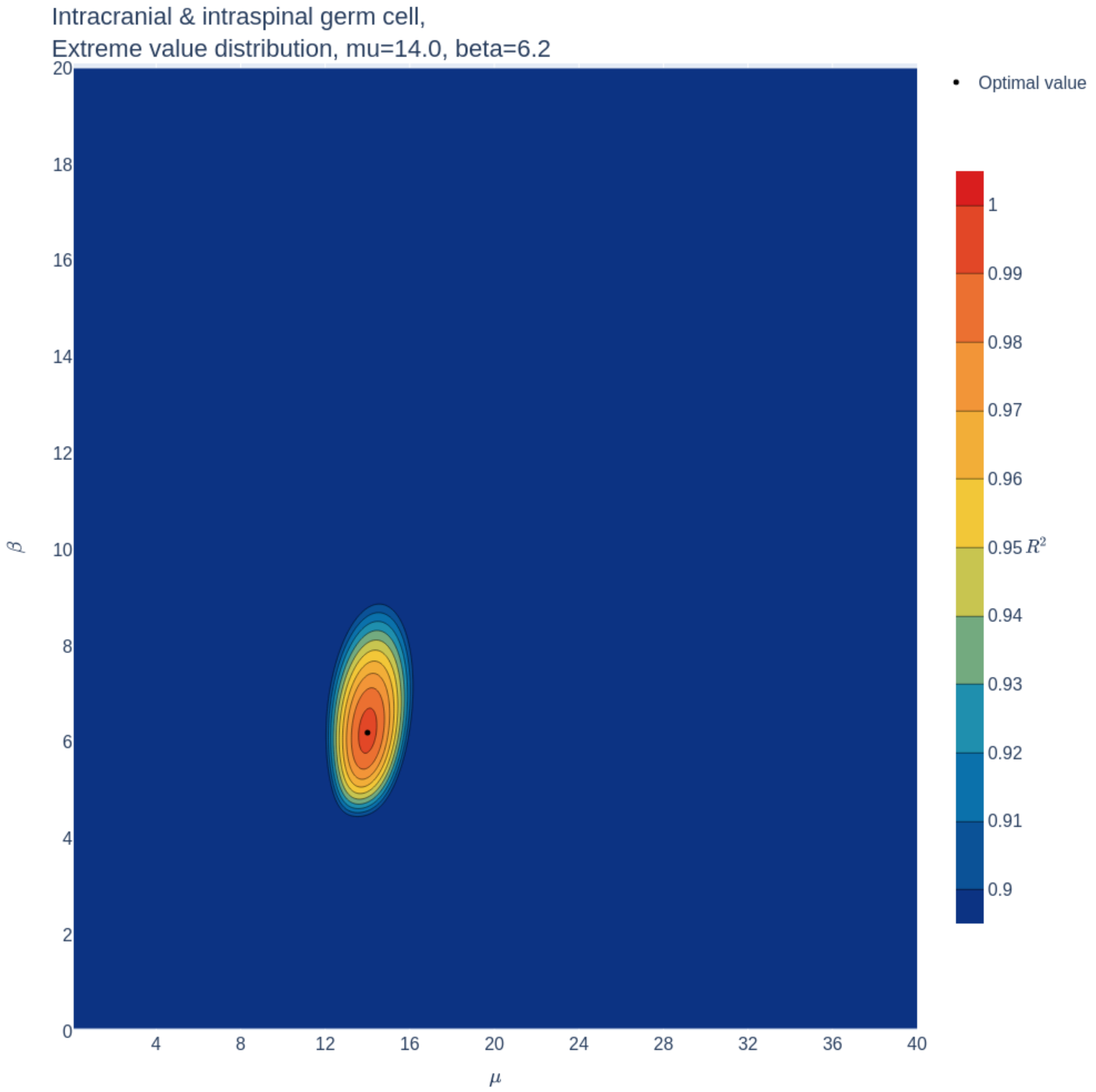

Web Figure 16. Goodness of fit of the extreme value distribution to the age distribution of incidence of intracranial and intraspinal germ cell tumors as a function of various parameter combinations.

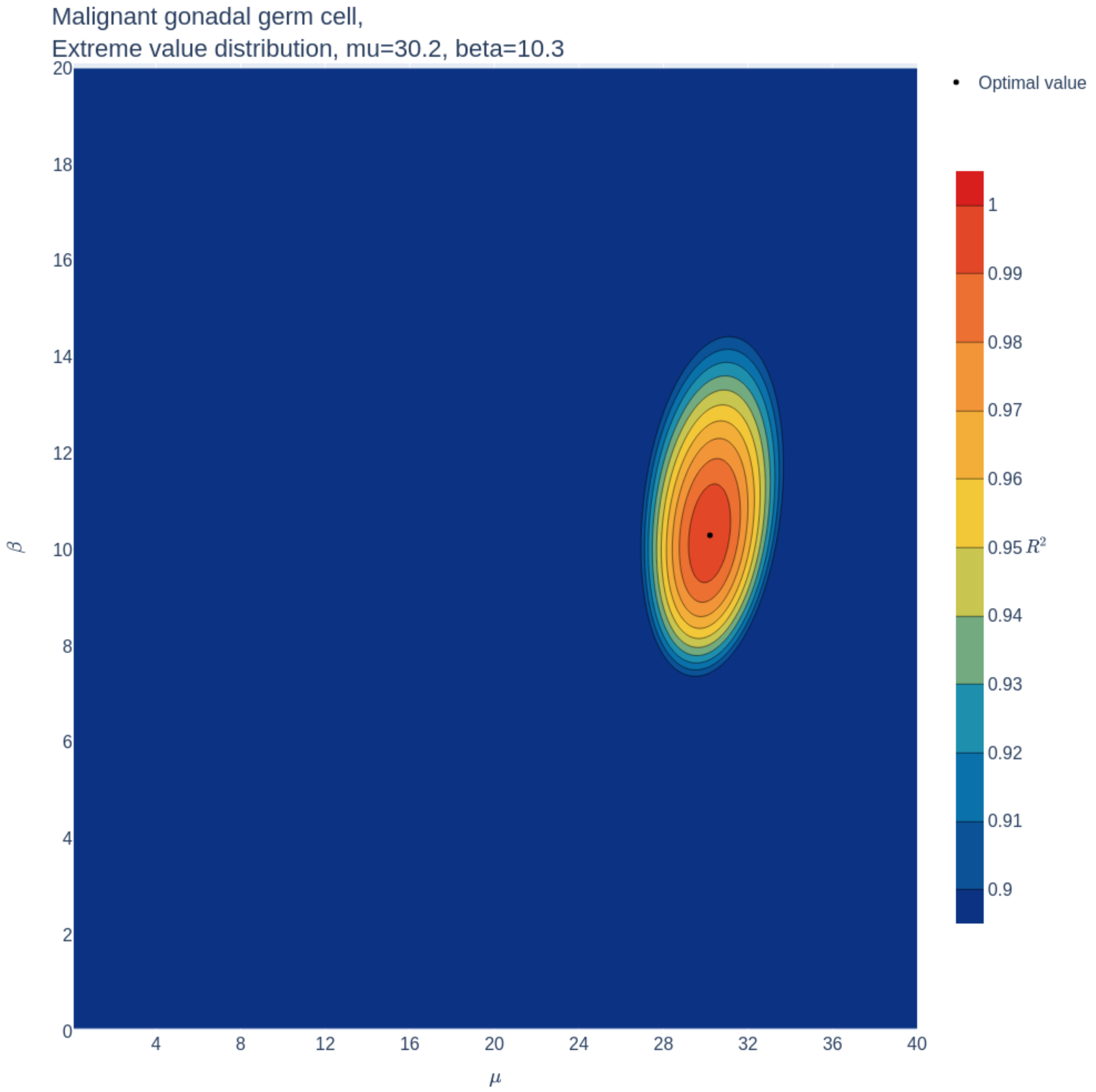

Web Figure 17. Goodness of fit of the extreme value distribution to the age distribution of incidence of malignant gonadal germ cell tumors as a function of various parameter combinations.

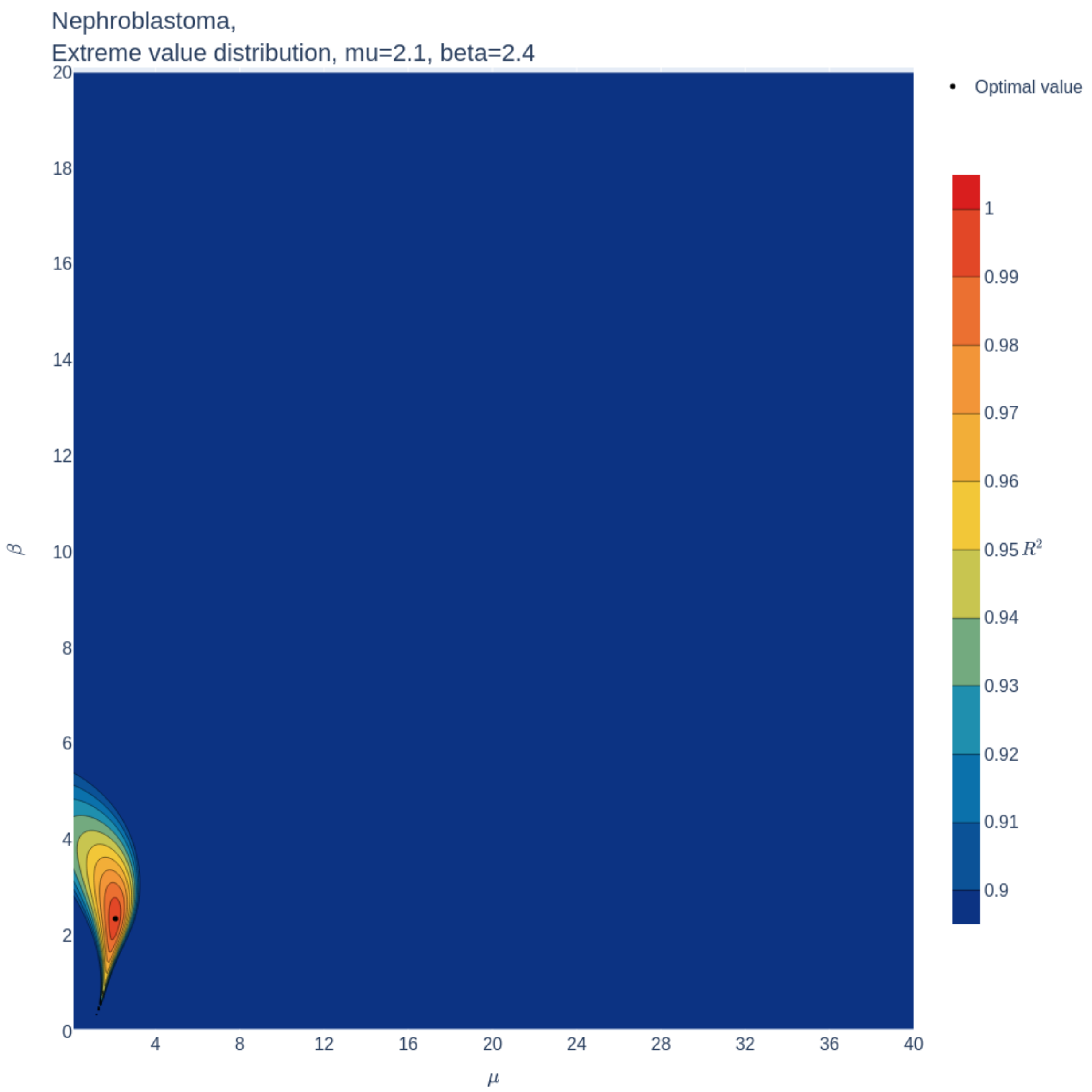

Web Figure 18. Goodness of fit of the extreme value distribution to the age distribution of incidence of nephroblastoma as a function of various parameter combinations.

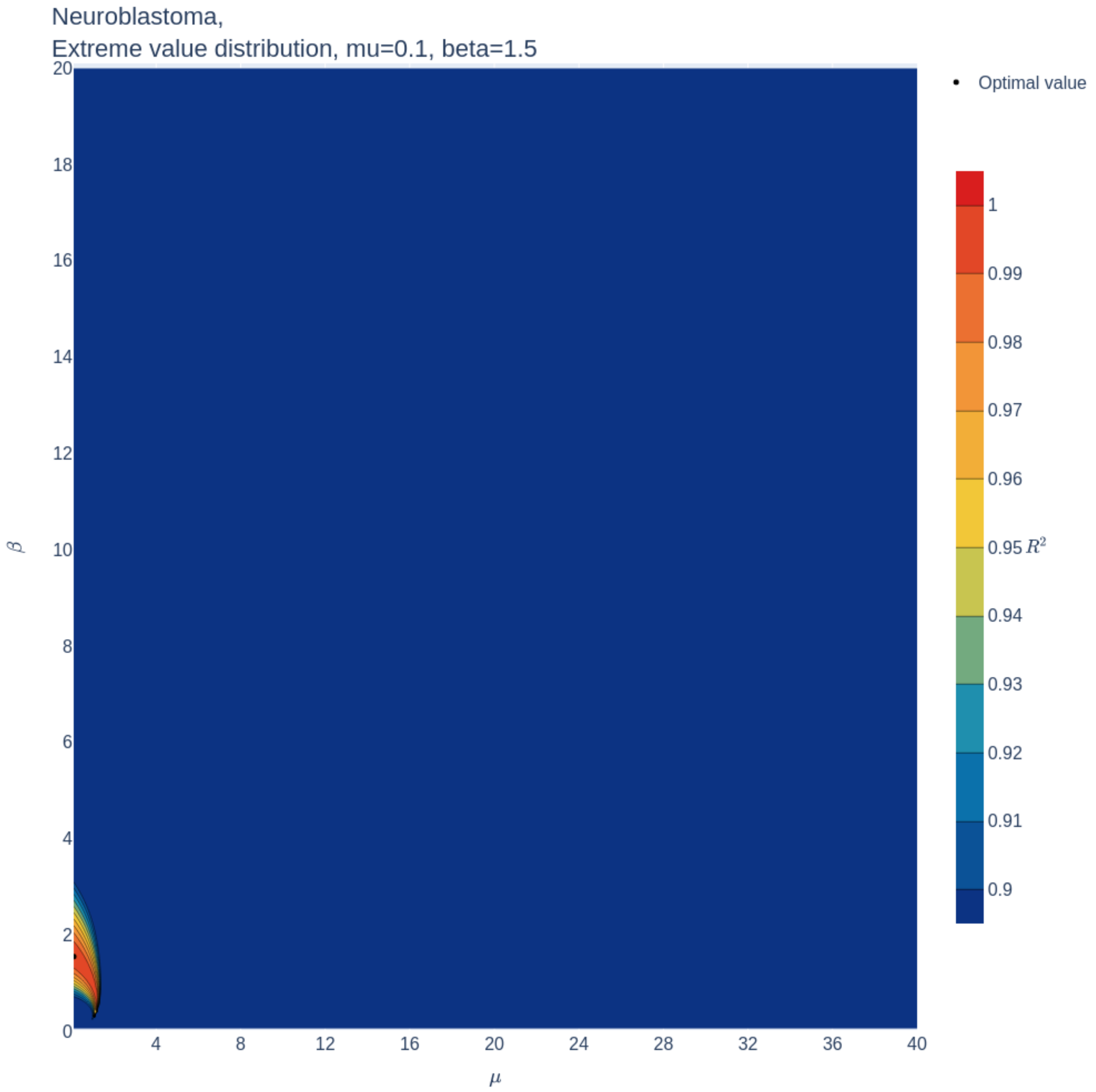

Web Figure 19. Goodness of fit of the extreme value distribution to the age distribution of incidence of neuroblastoma as a function of various parameter combinations.

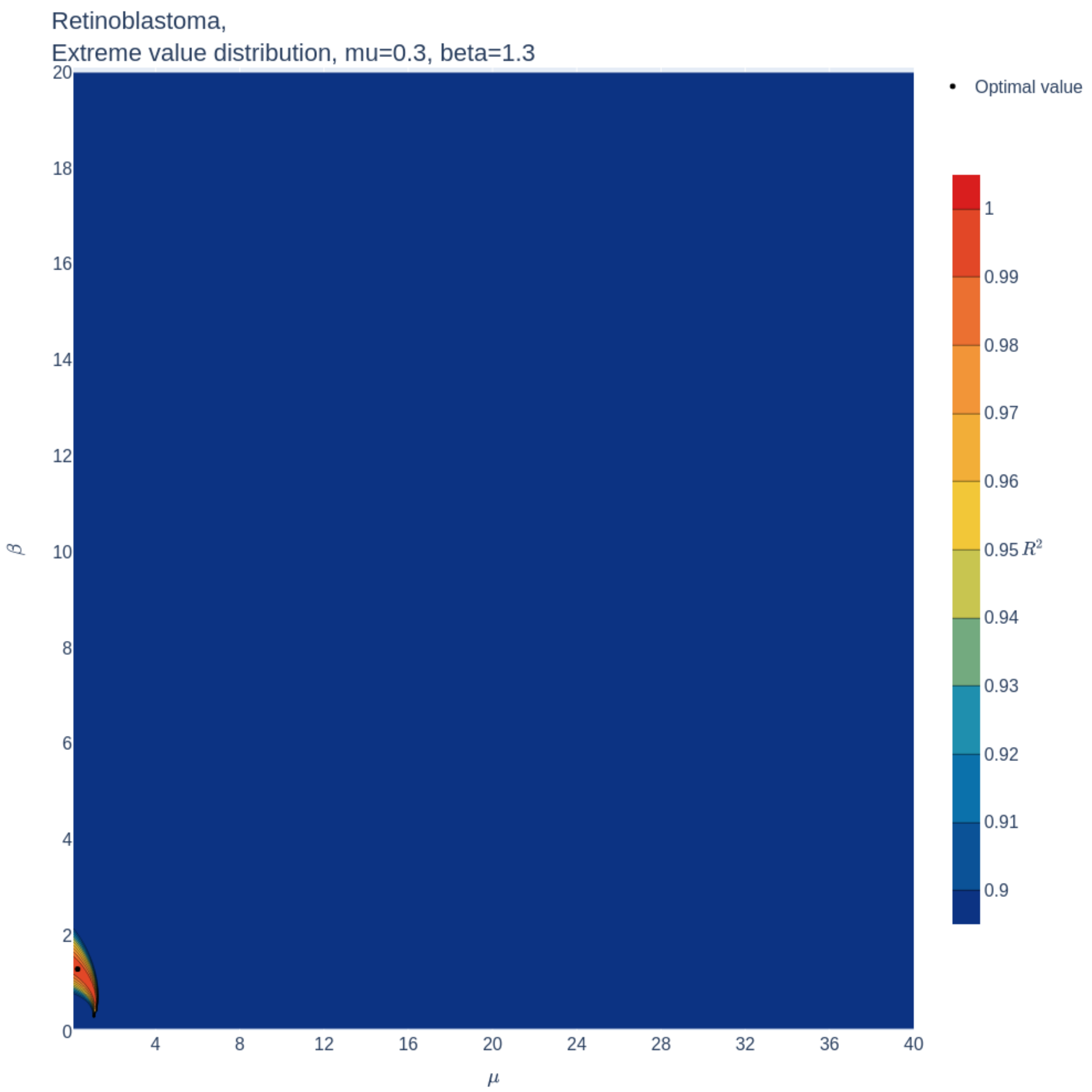

Web Figure 20. Goodness of fit of the extreme value distribution to the age distribution of incidence of retinoblastoma as a function of various parameter combinations.

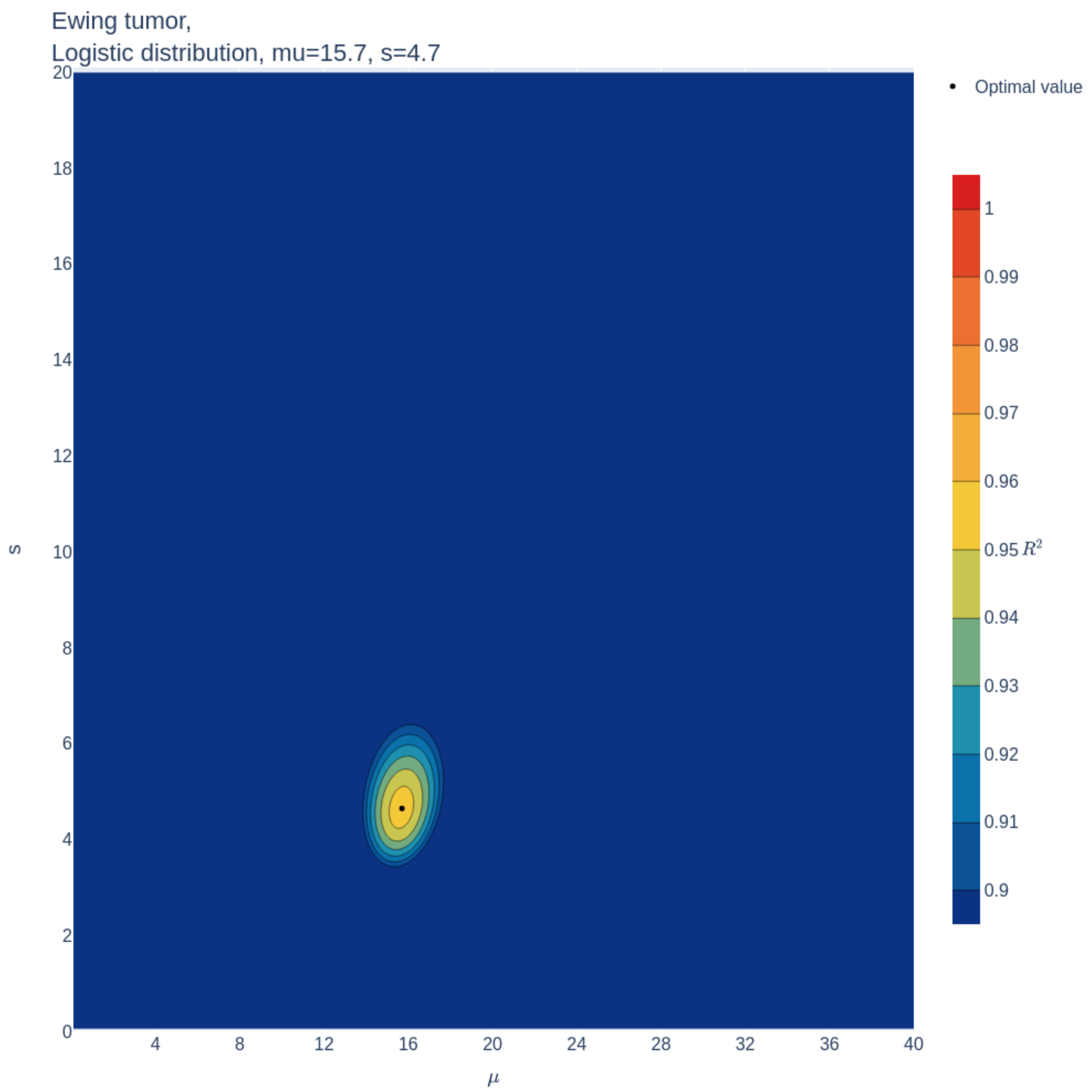

Web Figure 21. Goodness of fit of the logistic distribution to the age distribution of incidence of Ewing tumor as a function of various parameter combinations.

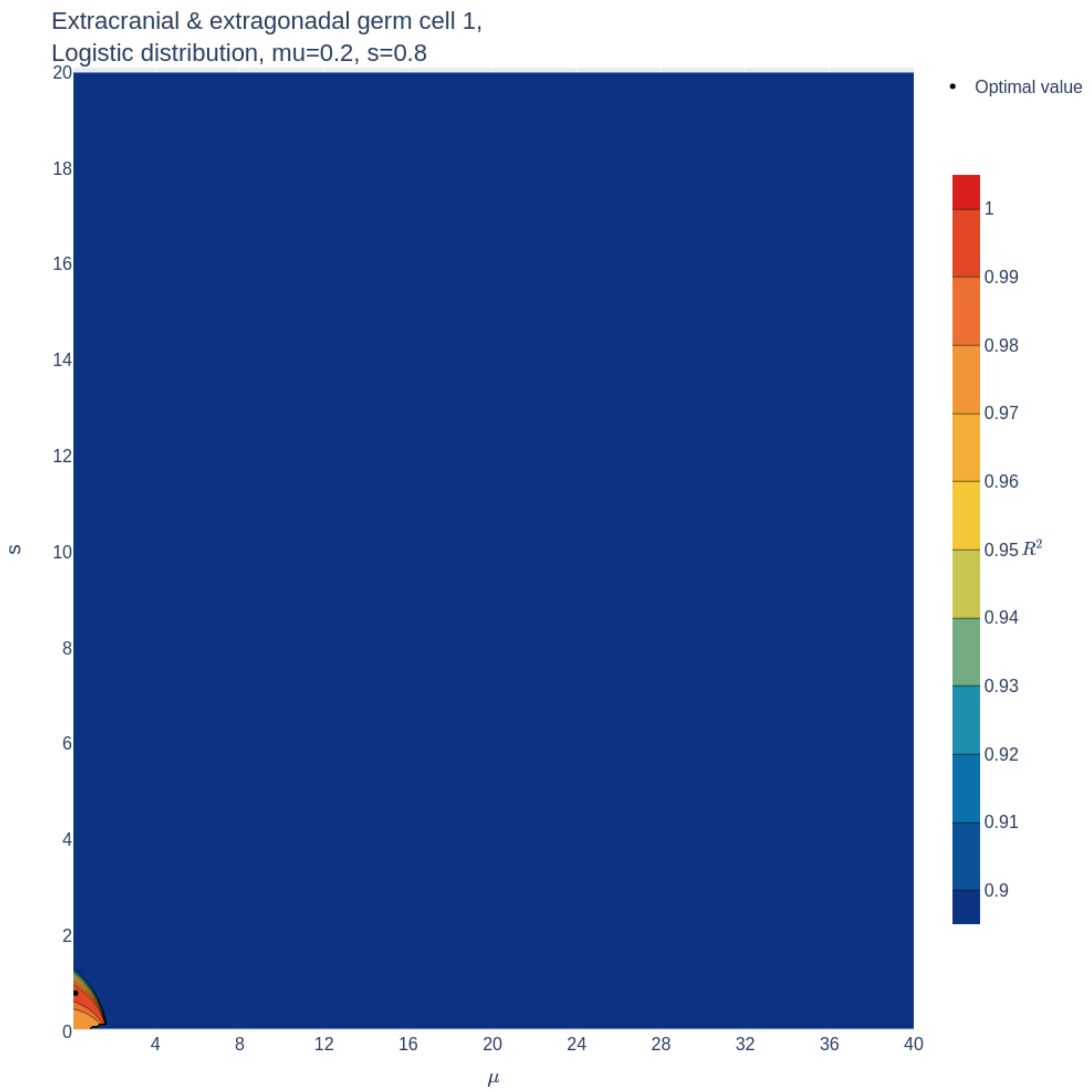

Web Figure 22. Goodness of fit of the logistic distribution to the age distribution of incidence of extracranial and extragonadal germ cell tumors of childhood as a function of various parameter combinations.

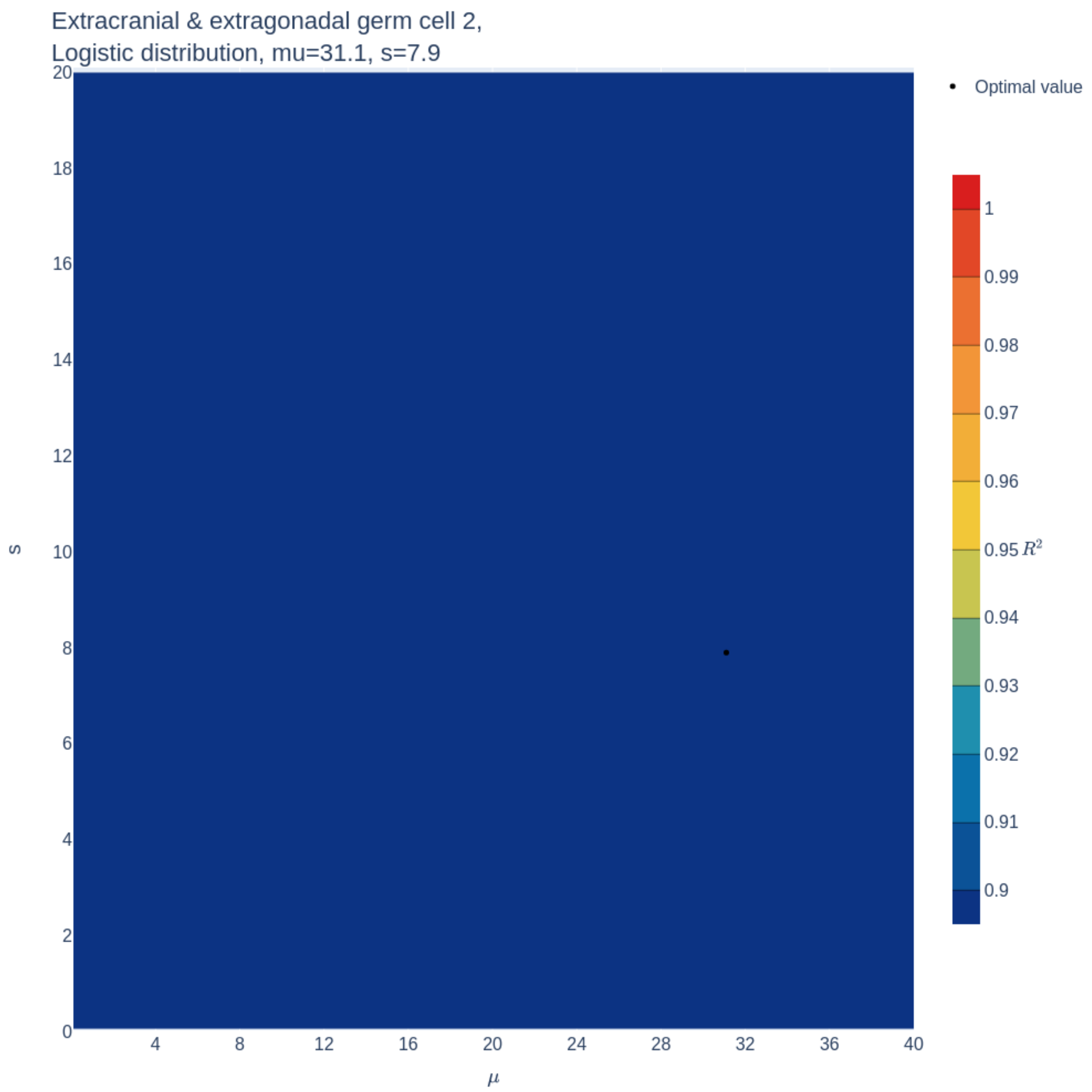

Web Figure 23. Goodness of fit of the logistic distribution to the age distribution of incidence of extracranial and extragonadal germ cell tumors of young adulthood as a function of various parameter combinations.

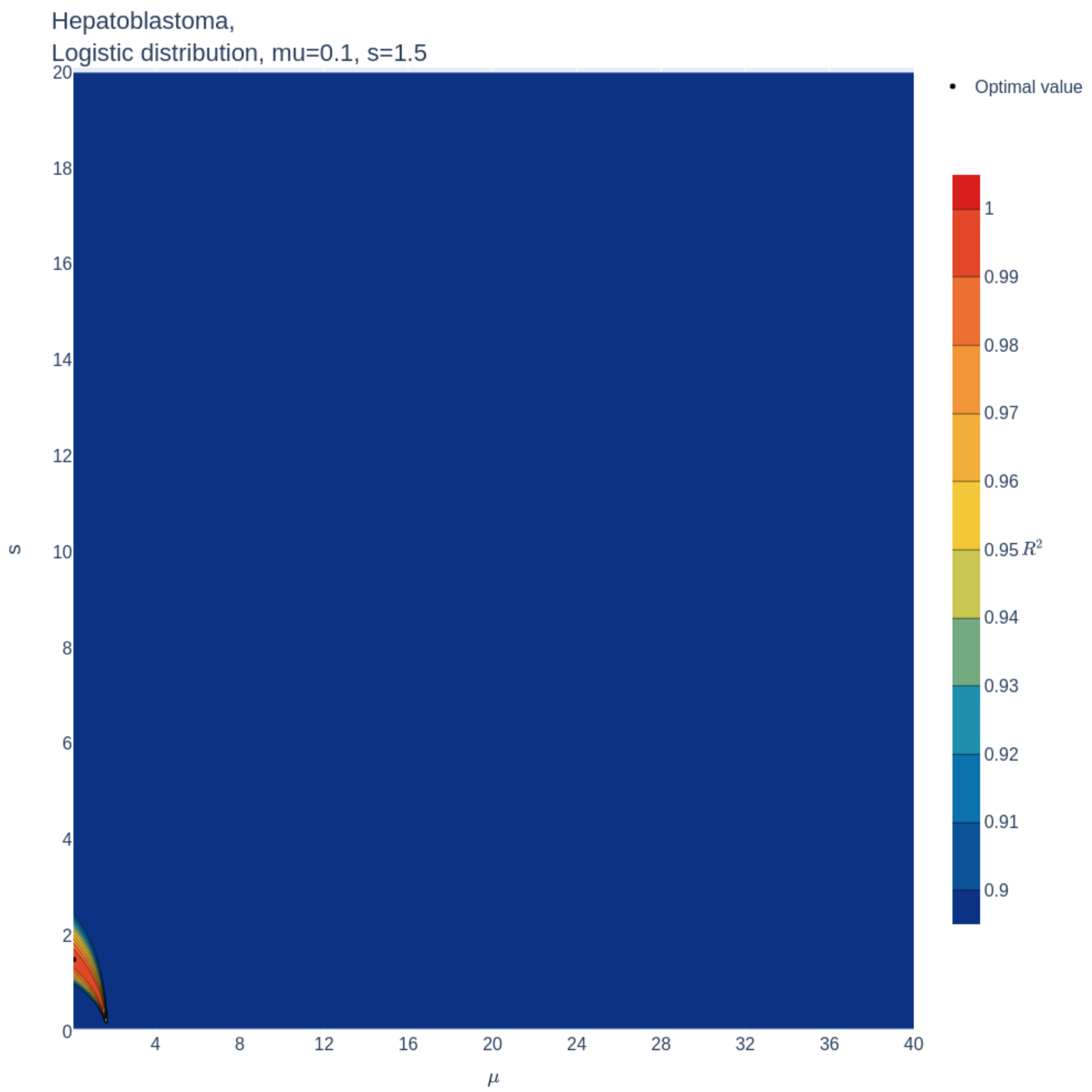

Web Figure 24. Goodness of fit of the logistic distribution to the age distribution of incidence of hepatoblastoma as a function of various parameter combinations.

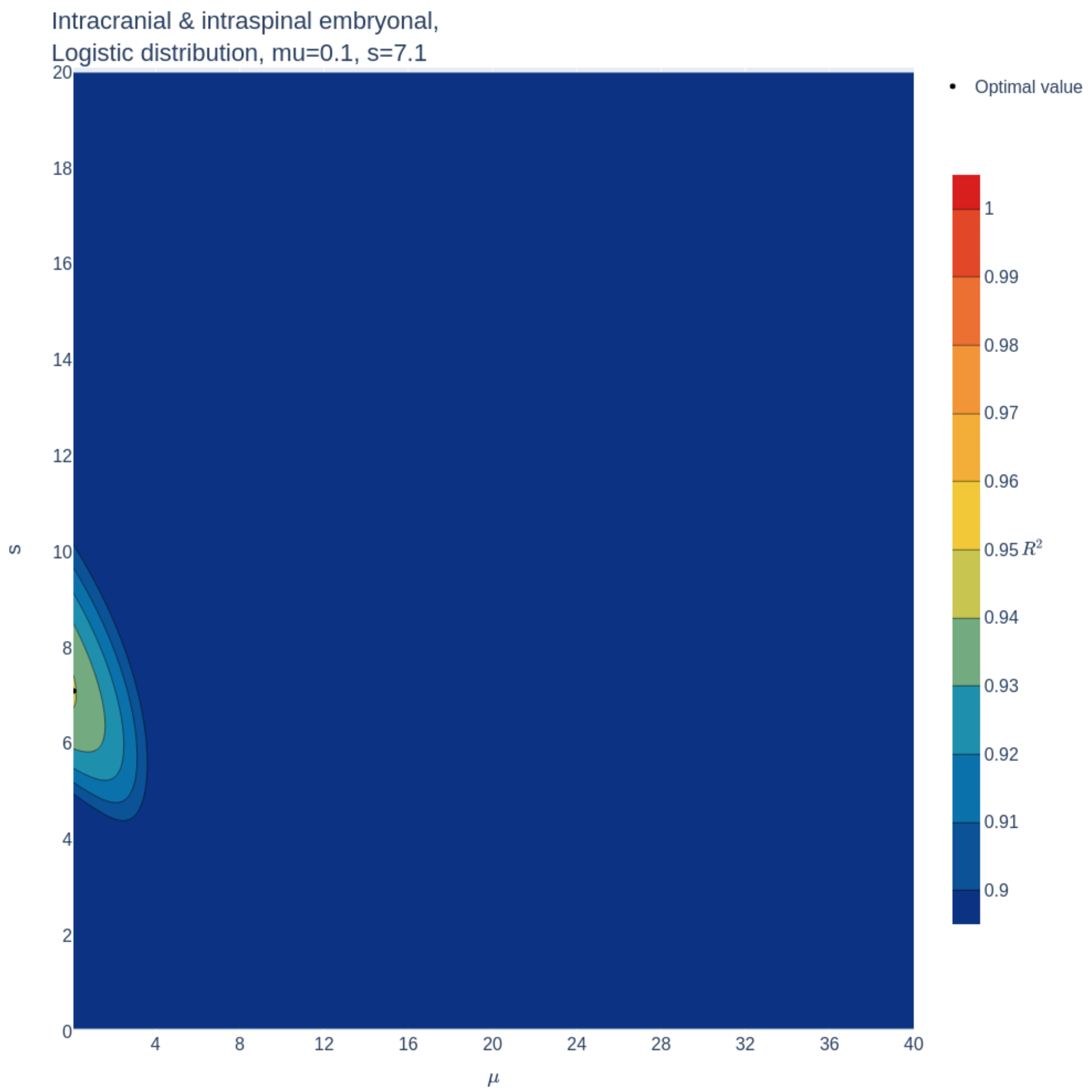

Web Figure 25. Goodness of fit of the logistic distribution to the age distribution of incidence of intracranial and intraspinal embryonal tumors as a function of various parameter combinations.

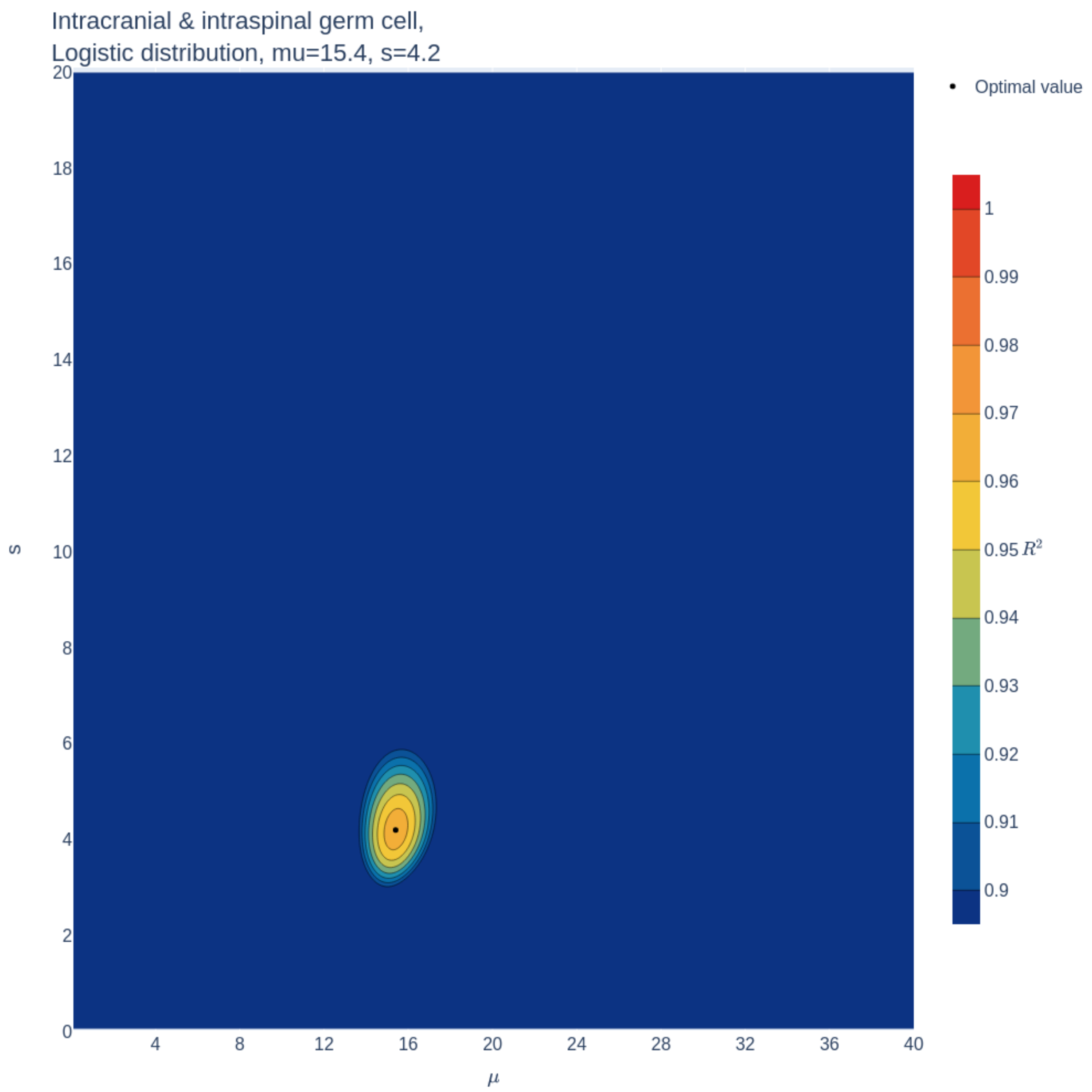

Web Figure 26. Goodness of fit of the logistic distribution to the age distribution of incidence of intracranial and intraspinal germ cell tumors as a function of various parameter combinations.

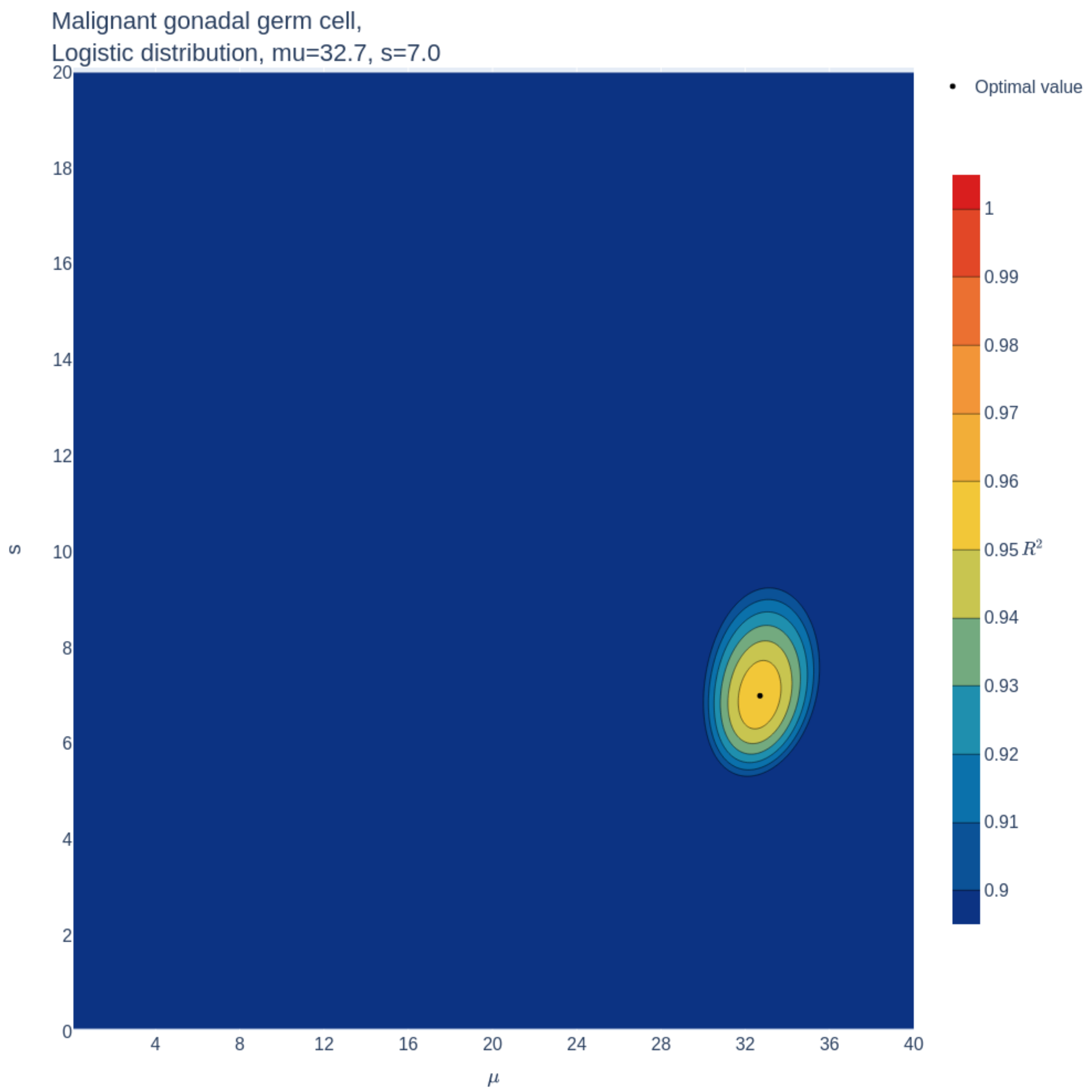

Web Figure 27. Goodness of fit of the logistic distribution to the age distribution of incidence of malignant gonadal germ cell tumors as a function of various parameter combinations.

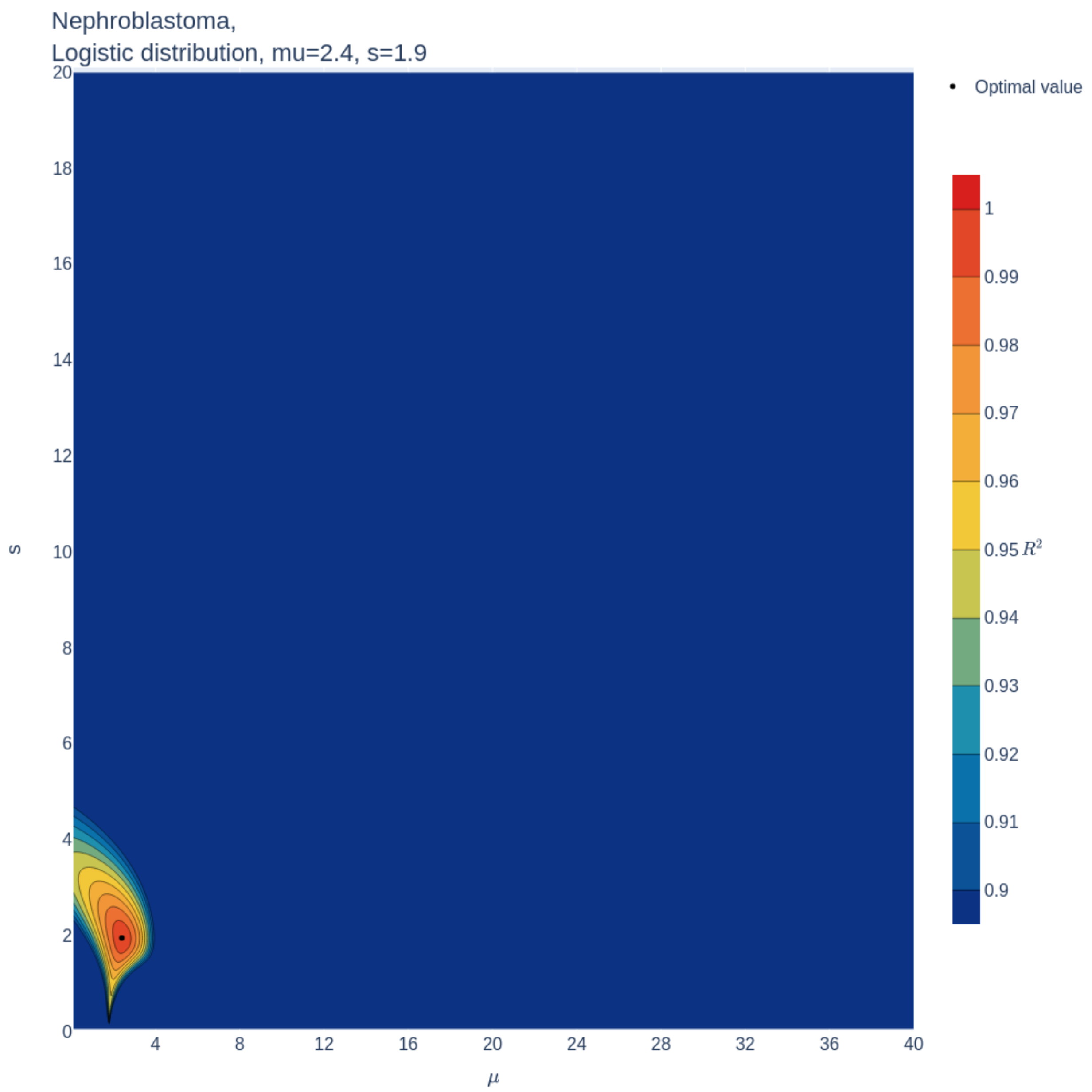

Web Figure 28. Goodness of fit of the logistic distribution to the age distribution of incidence of nephroblastoma as a function of various parameter combinations.

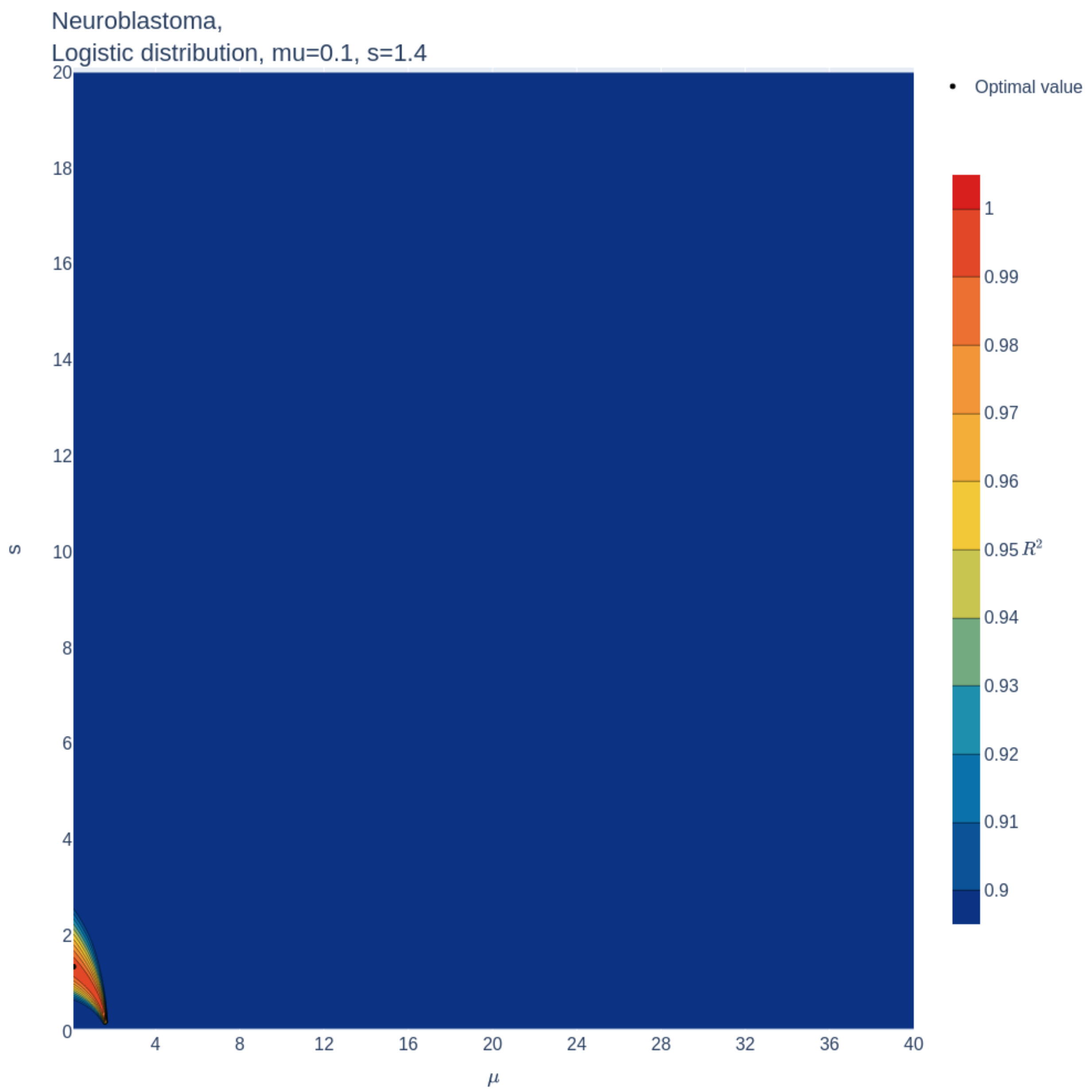

Web Figure 29. Goodness of fit of the logistic distribution to the age distribution of incidence of neuroblastoma as a function of various parameter combinations.

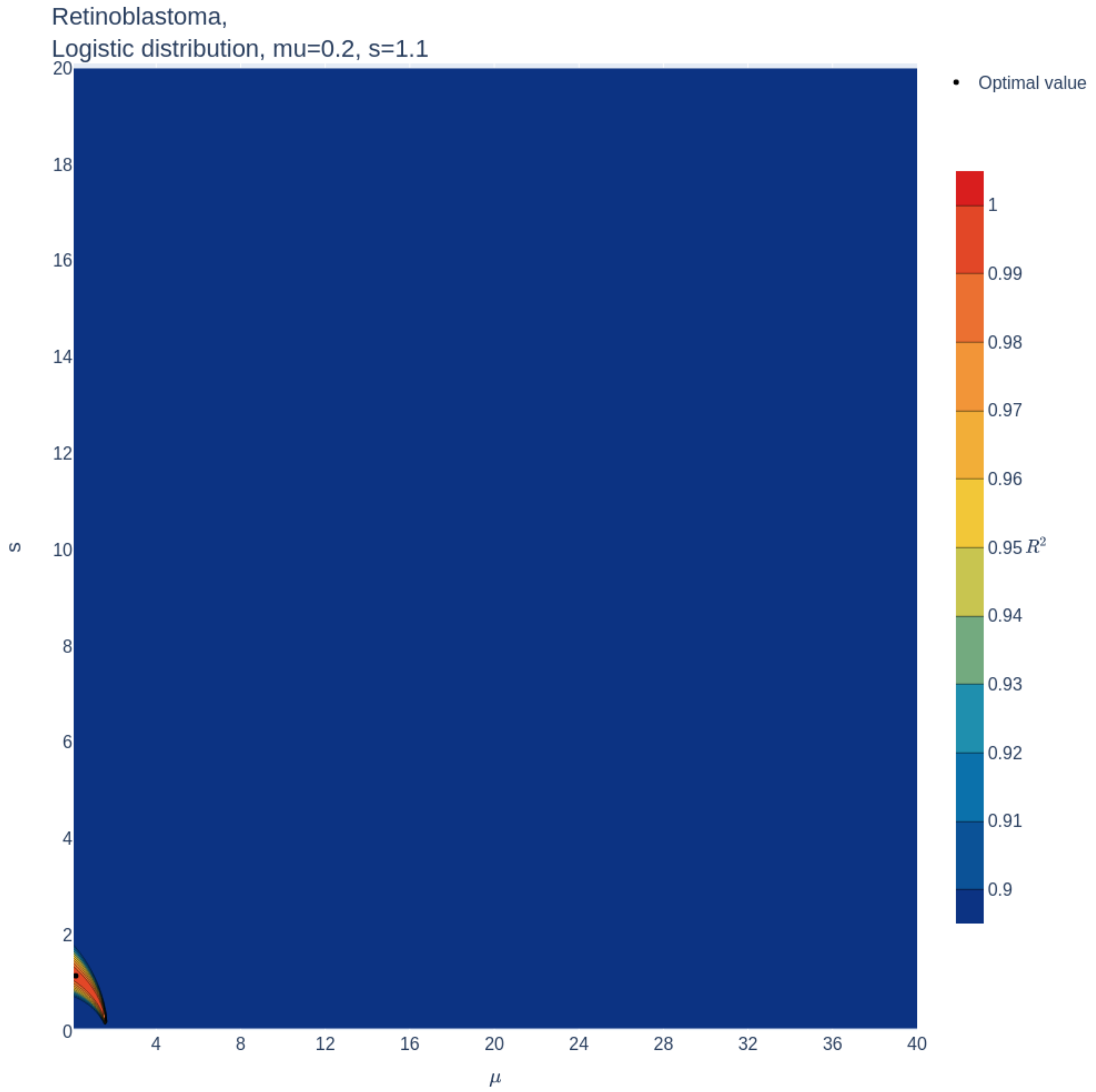

Web Figure 30. Goodness of fit of the logistic distribution to the age distribution of incidence of retinoblastoma as a function of various parameter combinations.

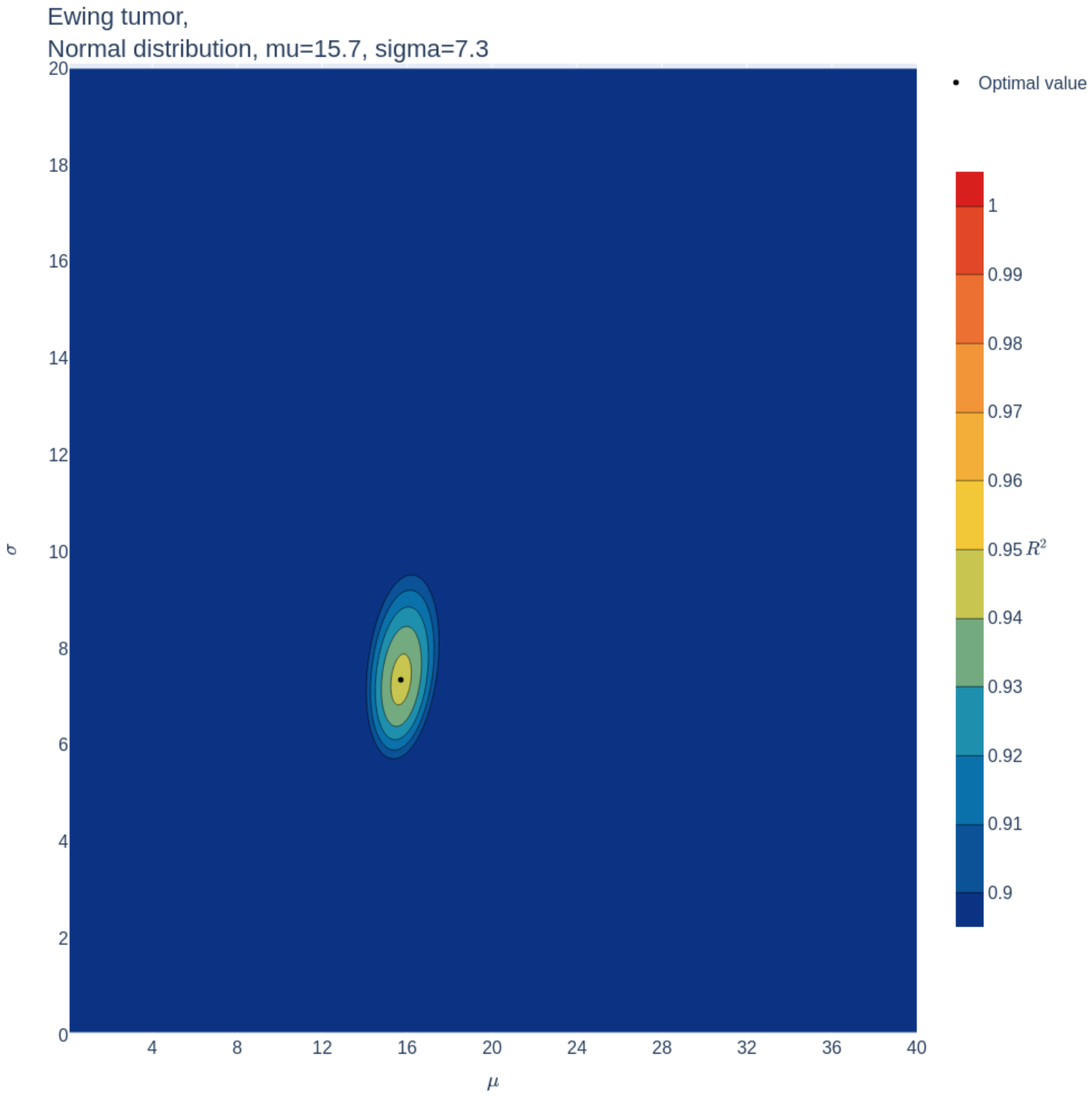

Web Figure 31. Goodness of fit of the normal distribution to the age distribution of incidence of Ewing tumor as a function of various parameter combinations.

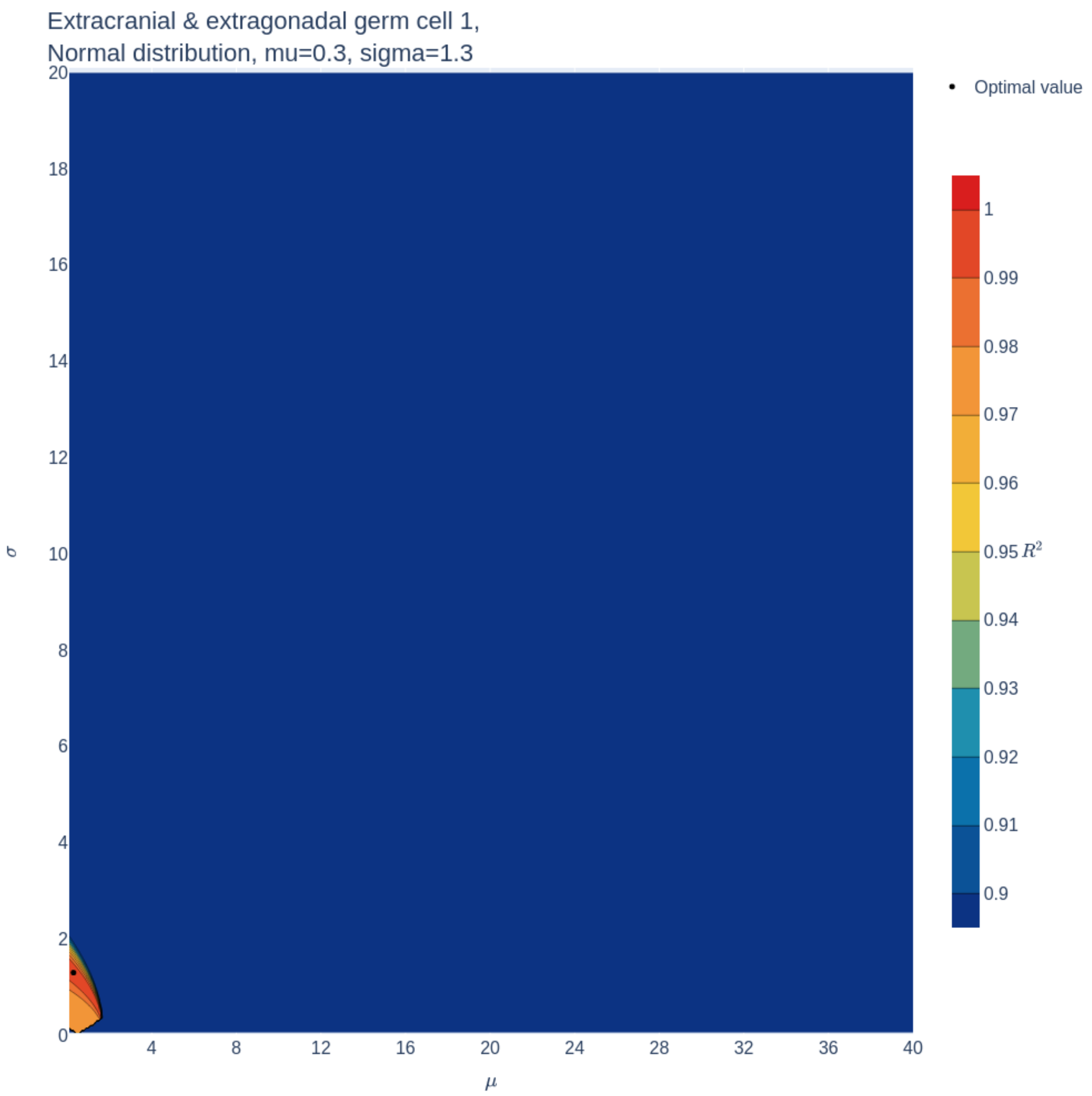

Web Figure 32. Goodness of fit of the normal distribution to the age distribution of incidence of extracranial and extragonadal germ cell tumors of childhood as a function of various parameter combinations.

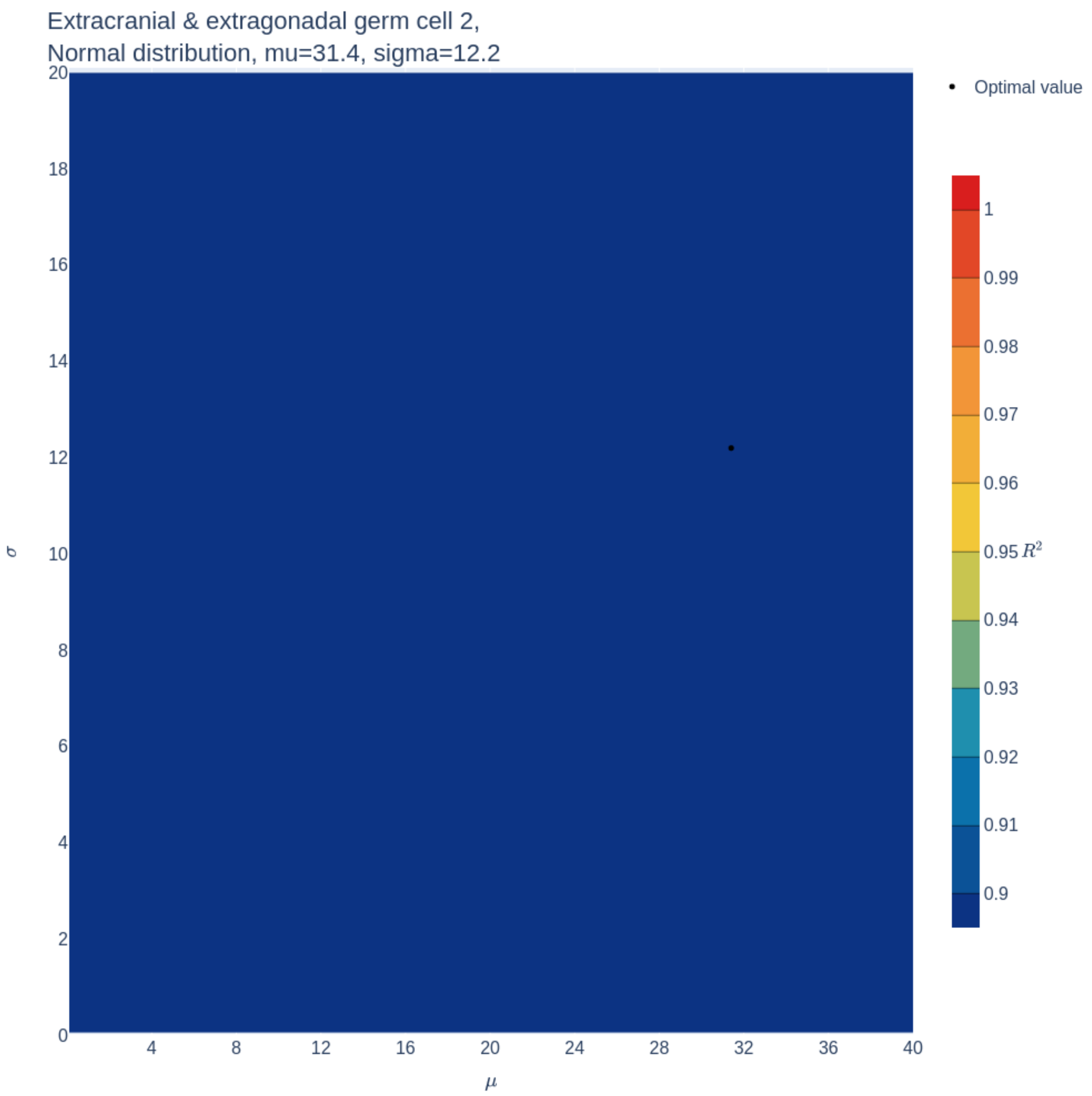

Web Figure 33. Goodness of fit of the normal distribution to the age distribution of incidence of extracranial and extragonadal germ cell tumors of young adulthood as a function of various parameter combinations.

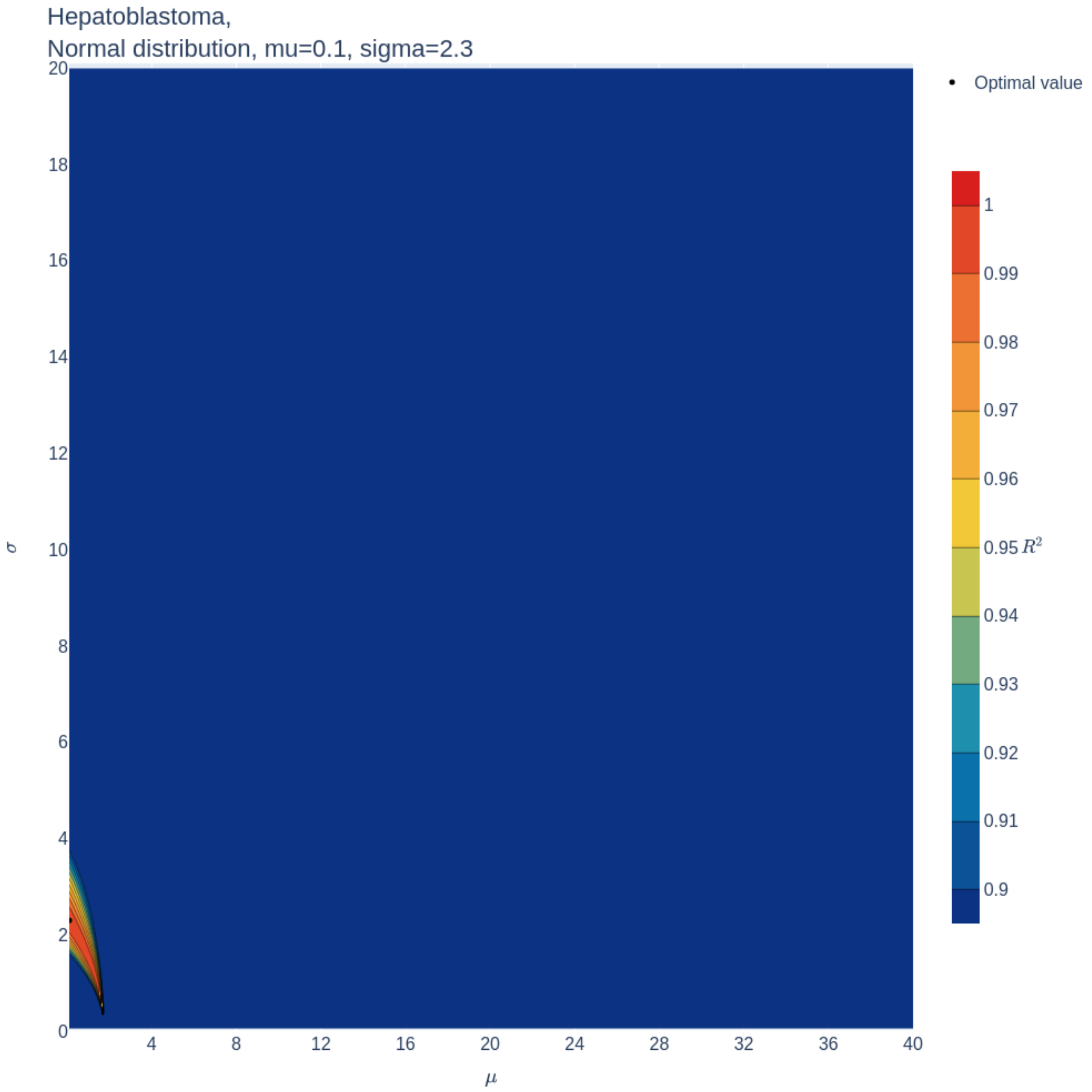

Web Figure 34. Goodness of fit of the normal distribution to the age distribution of incidence of hepatoblastoma as a function of various parameter combinations.

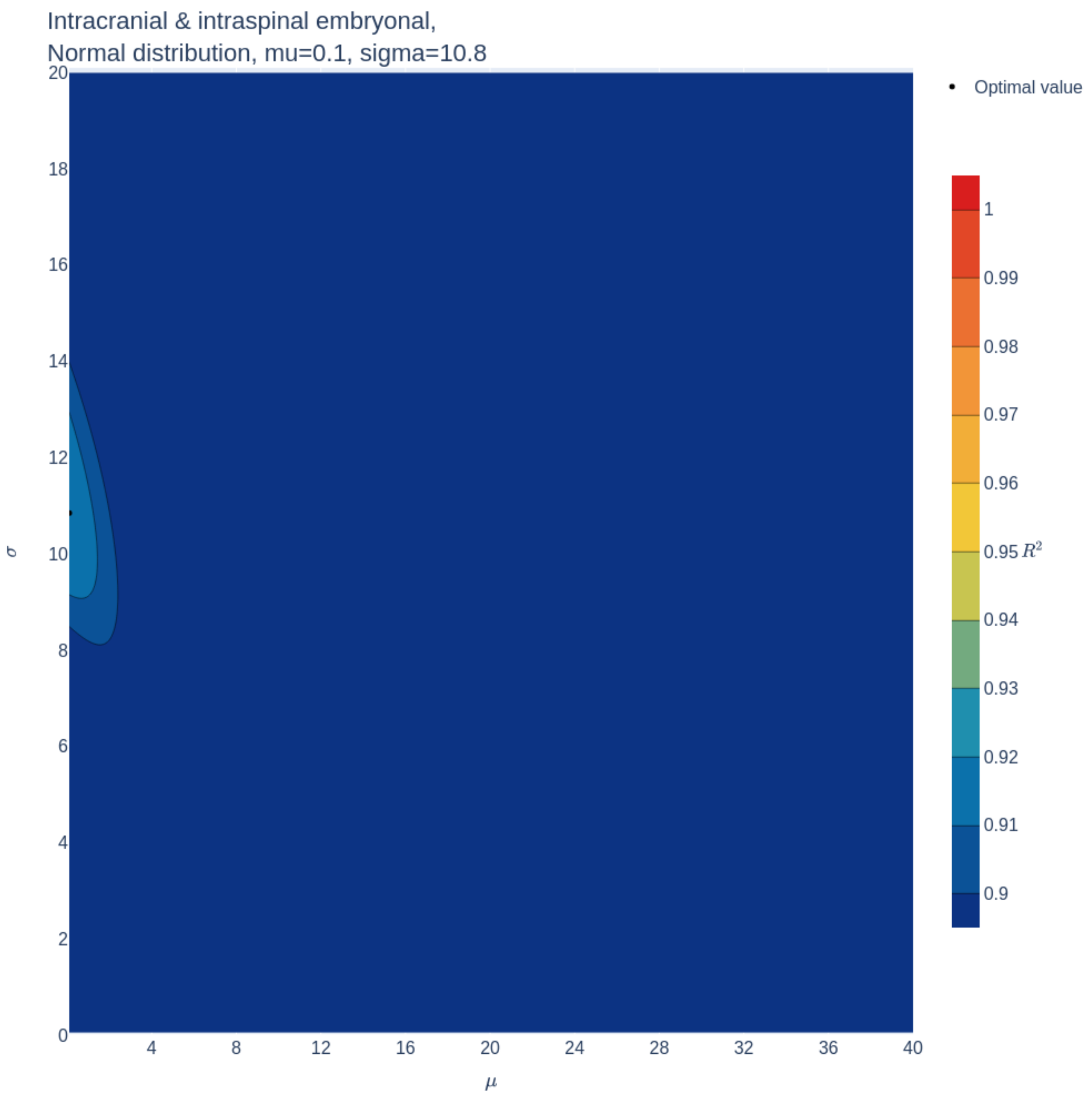

Web Figure 35. Goodness of fit of the normal distribution to the age distribution of incidence of intracranial and intraspinal embryonal tumors as a function of various parameter combinations.

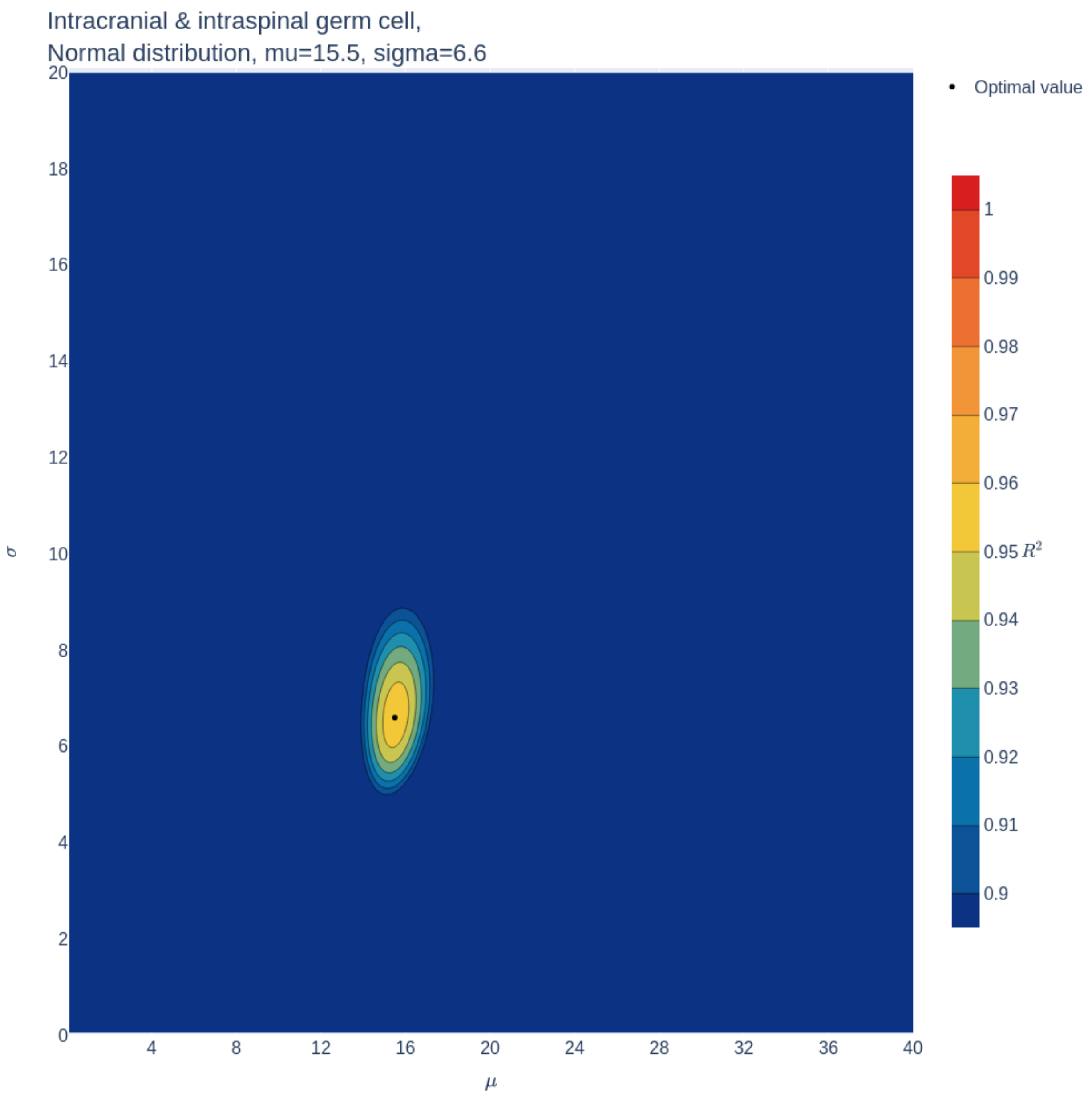

Web Figure 36. Goodness of fit of the normal distribution to the age distribution of incidence of intracranial and intraspinal germ cell tumors as a function of various parameter combinations.

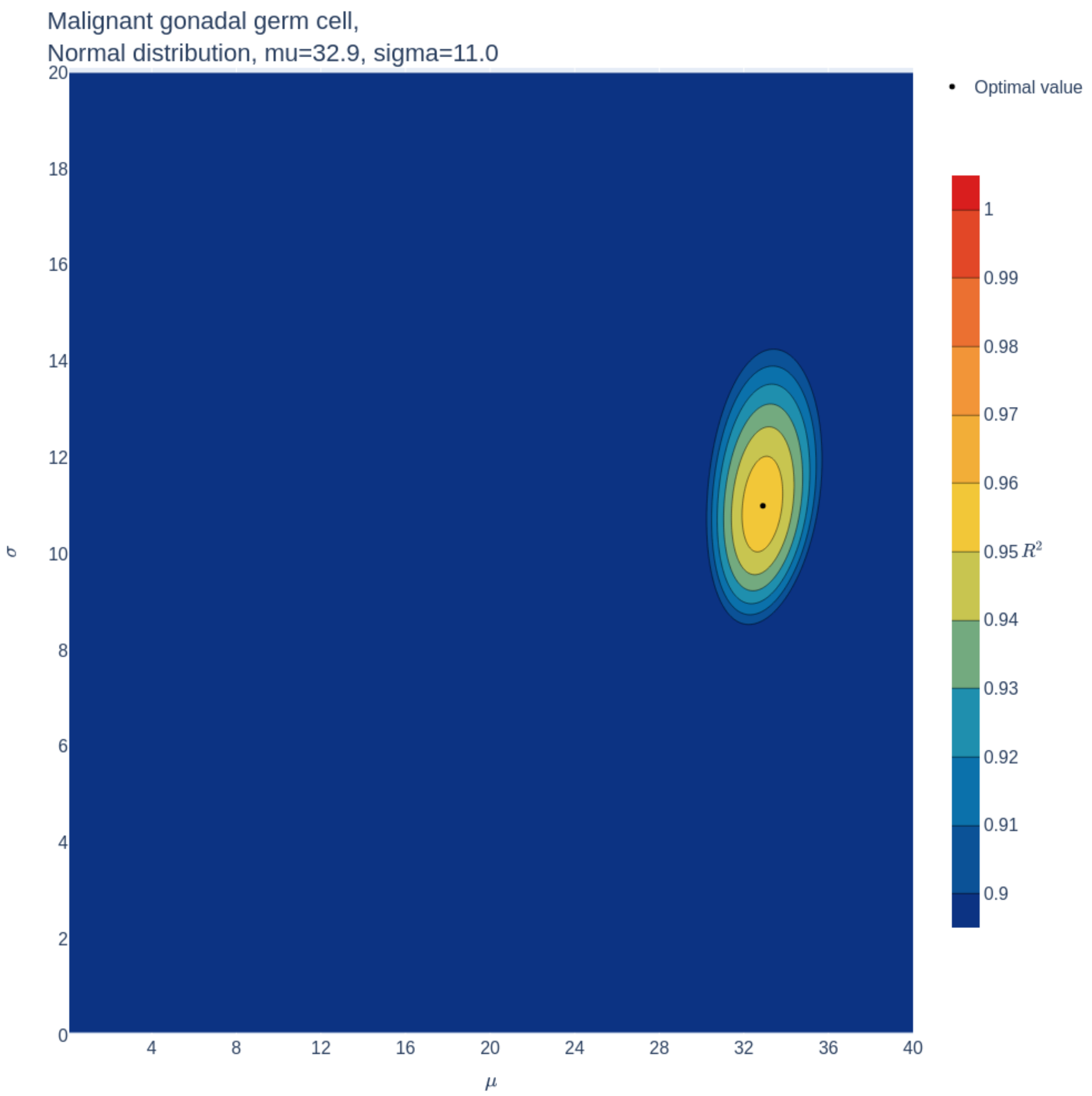

Web Figure 37. Goodness of fit of the normal distribution to the age distribution of incidence of malignant gonadal germ cell tumors as a function of various parameter combinations.

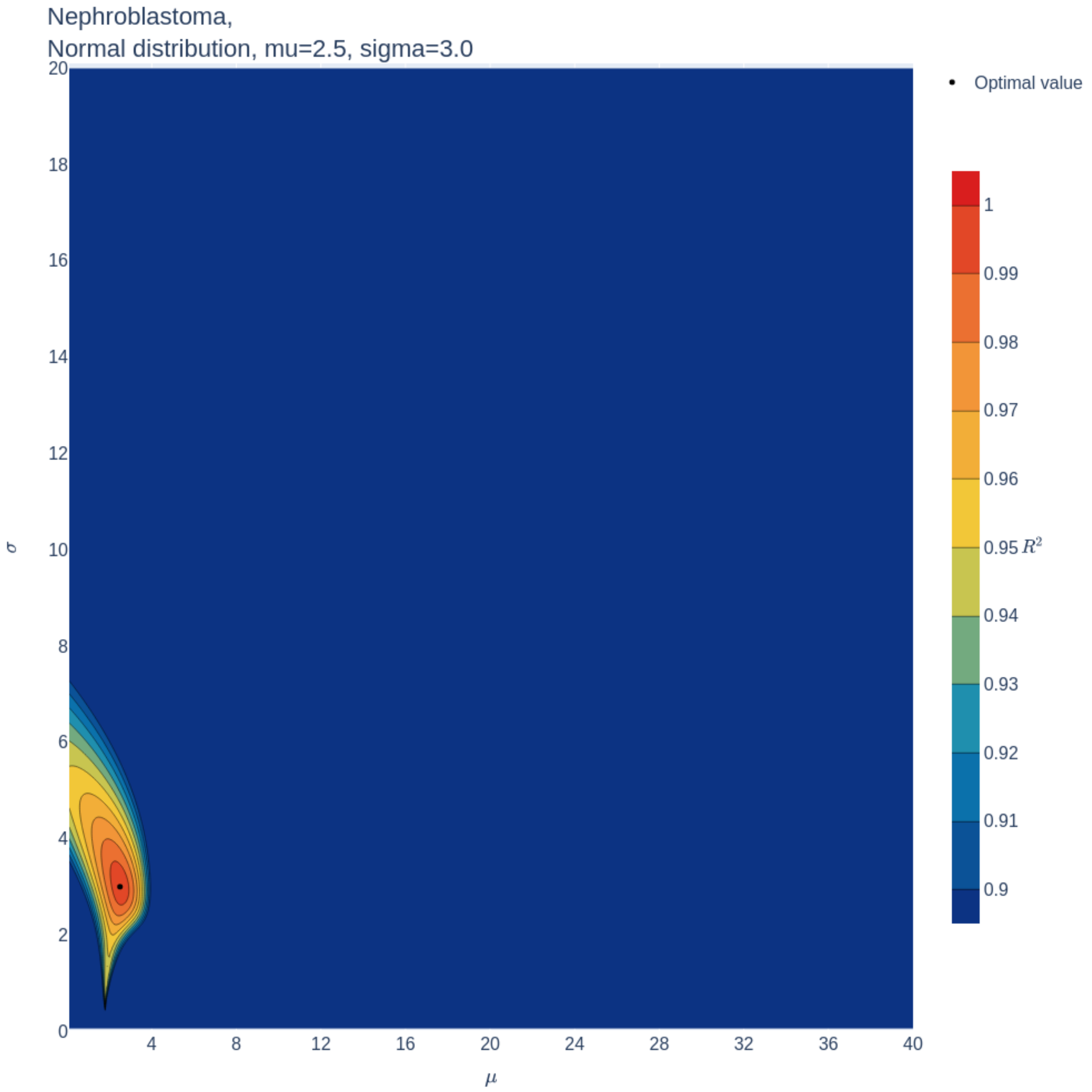

Web Figure 38. Goodness of fit of the normal distribution to the age distribution of incidence of nephroblastoma as a function of various parameter combinations.

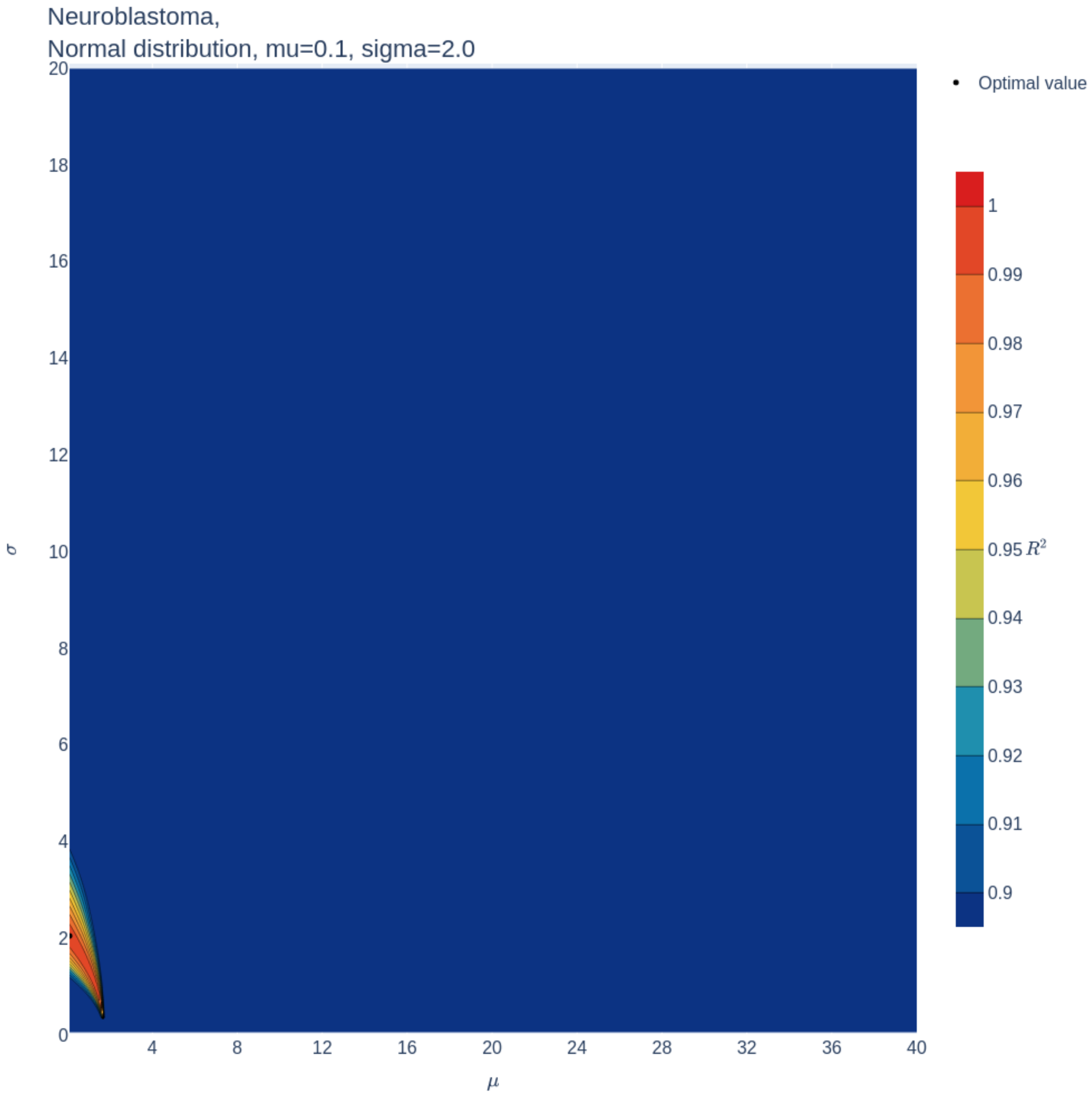

Web Figure 39. Goodness of fit of the normal distribution to the age distribution of incidence of neuroblastoma as a function of various parameter combinations.

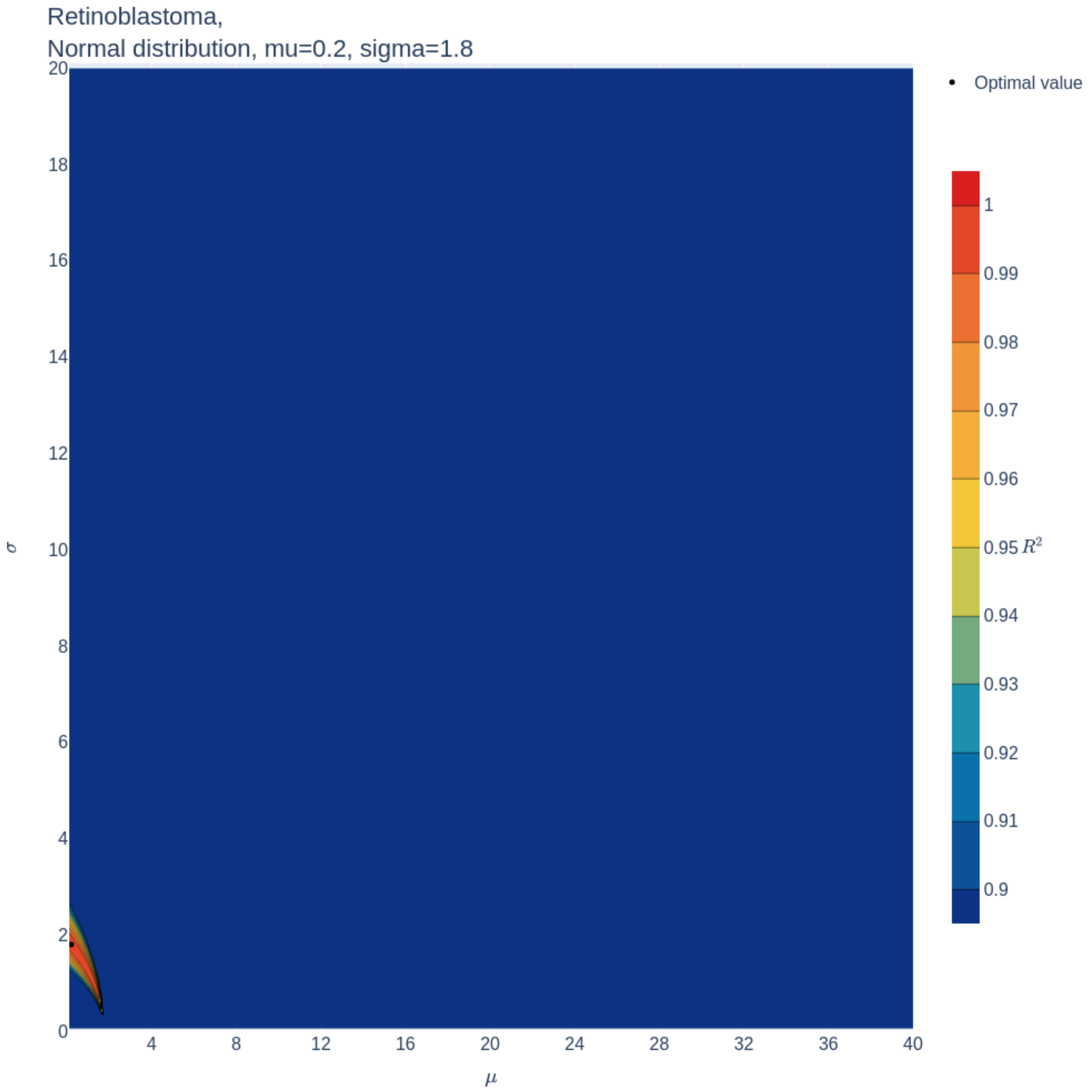

Web Figure 40. Goodness of fit of the normal distribution to the age distribution of incidence of retinoblastoma as a function of various parameter combinations.

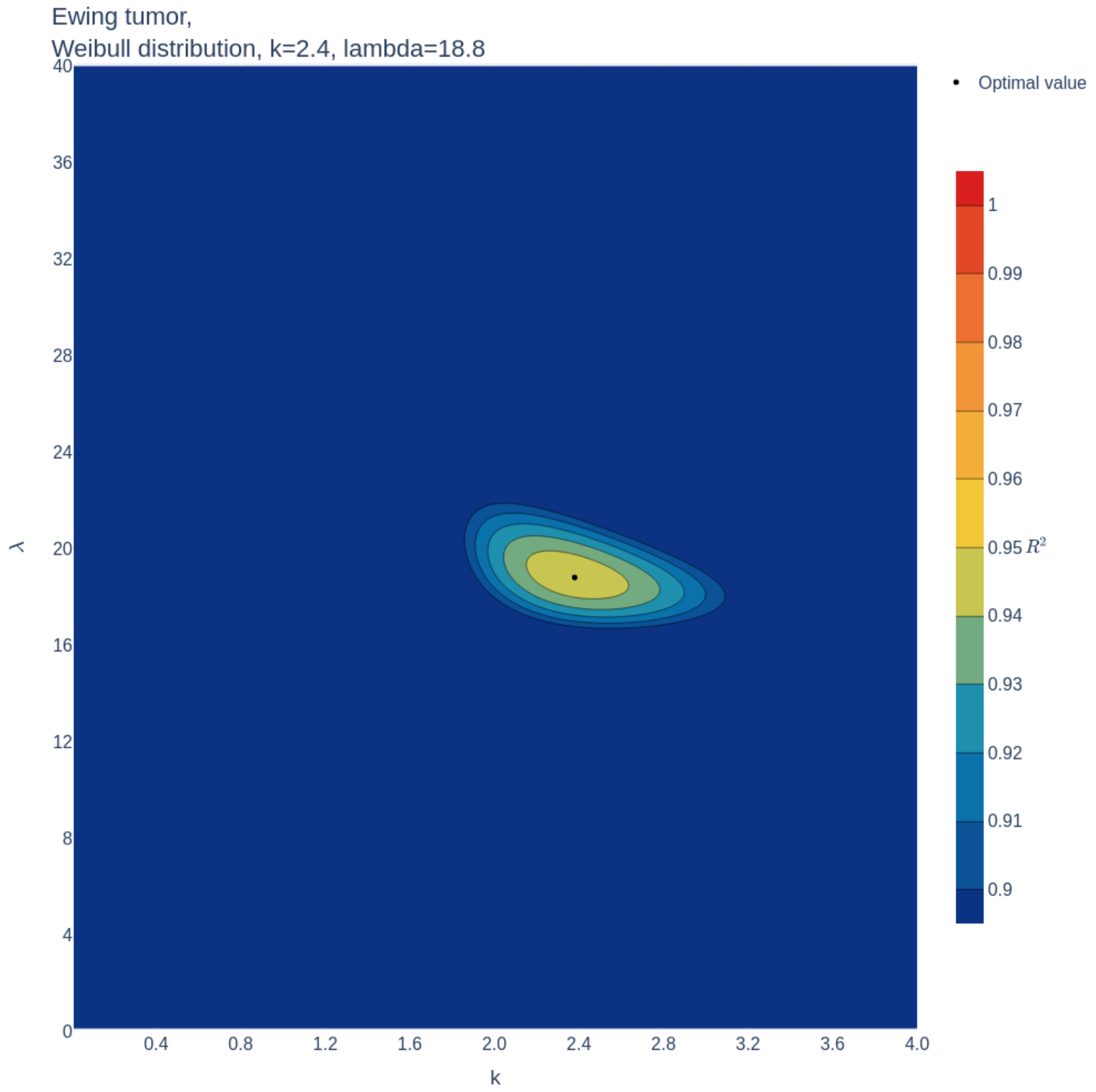

Web Figure 41. Goodness of fit of the Weibull distribution to the age distribution of incidence of Ewing tumor as a function of various parameter combinations.

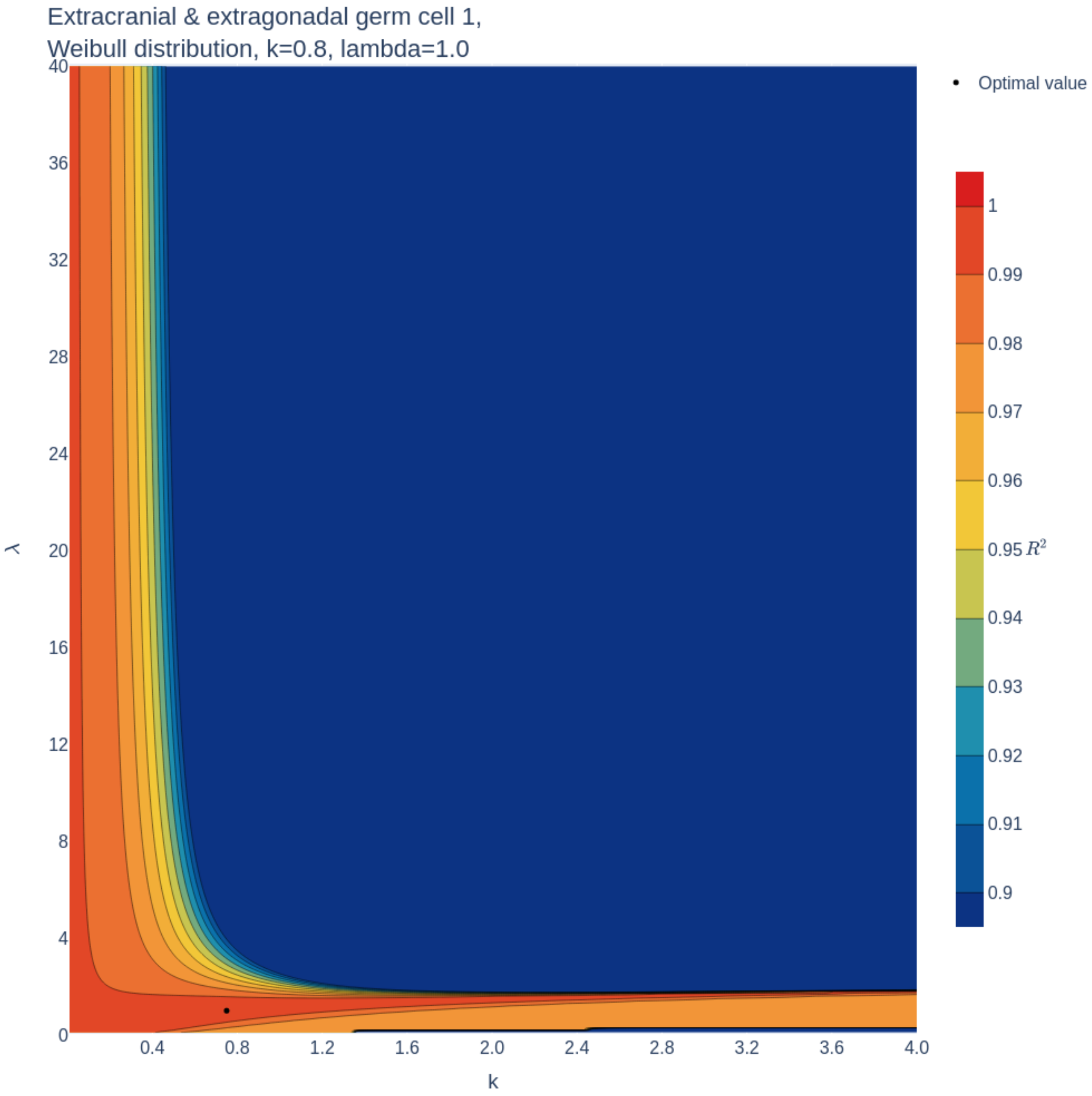

Web Figure 42. Goodness of fit of the Weibull distribution to the age distribution of incidence of extracranial and extragonadal germ cell tumors of childhood as a function of various parameter combinations.

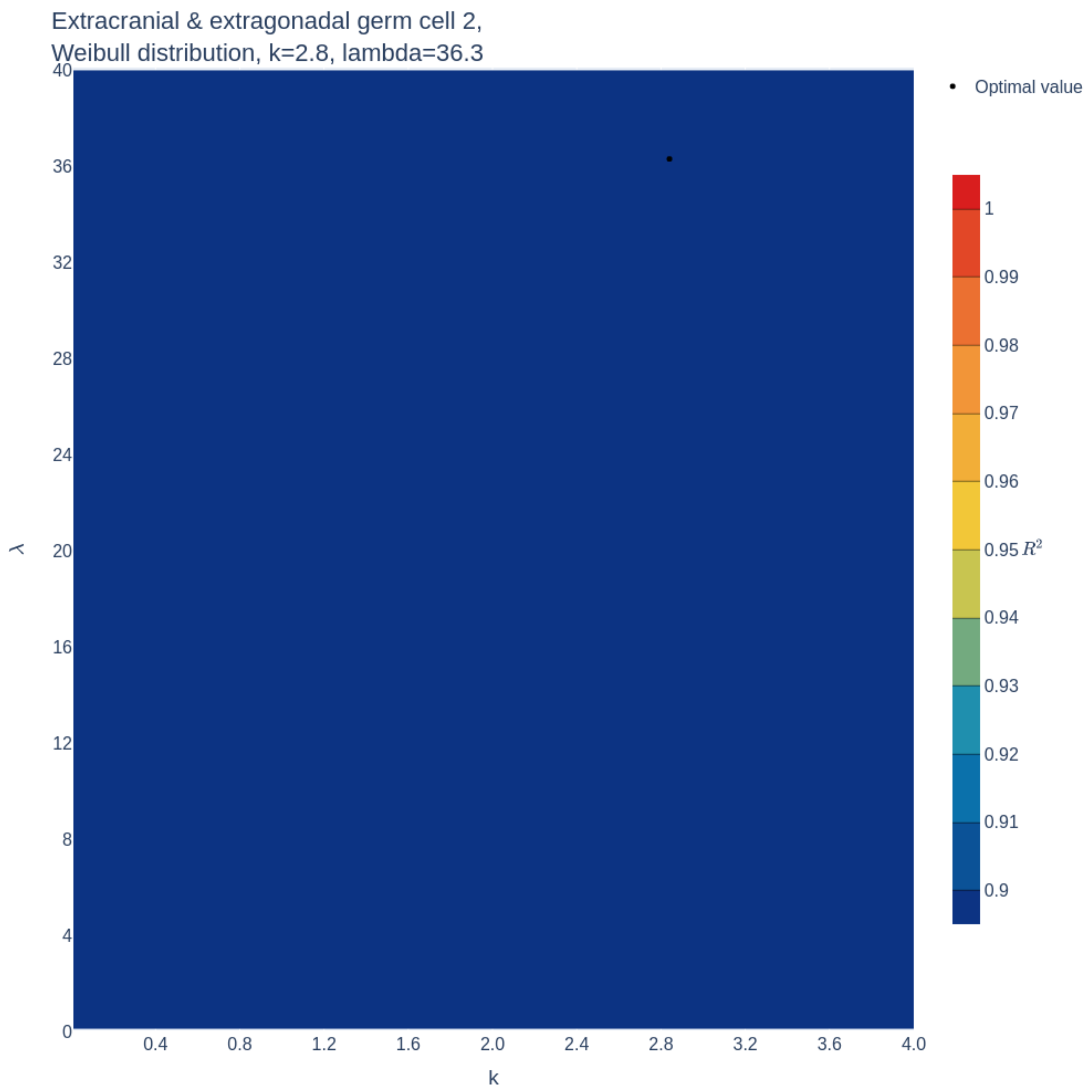

Web Figure 43. Goodness of fit of the Weibull distribution to the age distribution of incidence of extracranial and extragonadal germ cell tumors of young adulthood as a function of various parameter combinations.

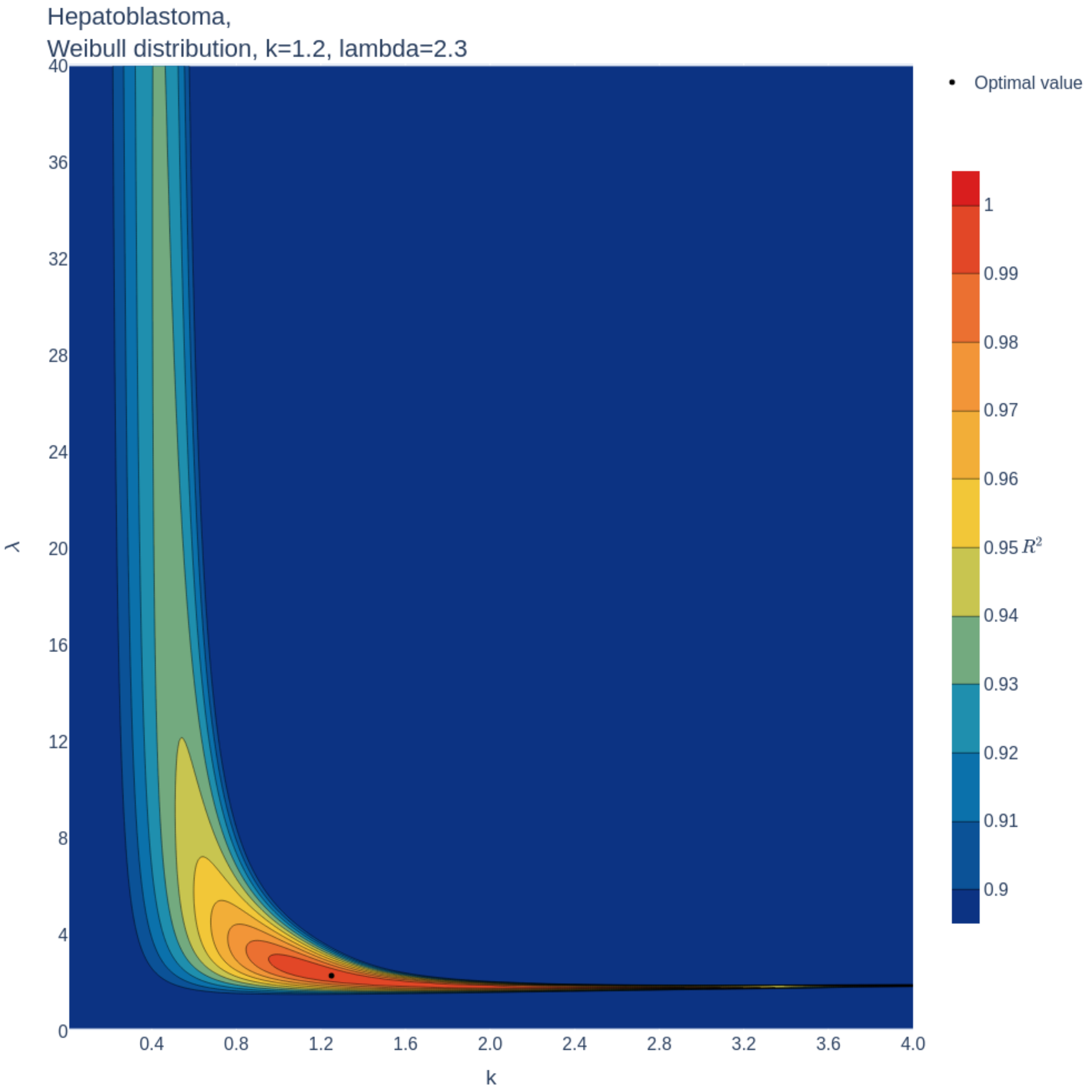

Web Figure 44. Goodness of fit of the Weibull distribution to the age distribution of incidence of hepatoblastoma as a function of various parameter combinations.

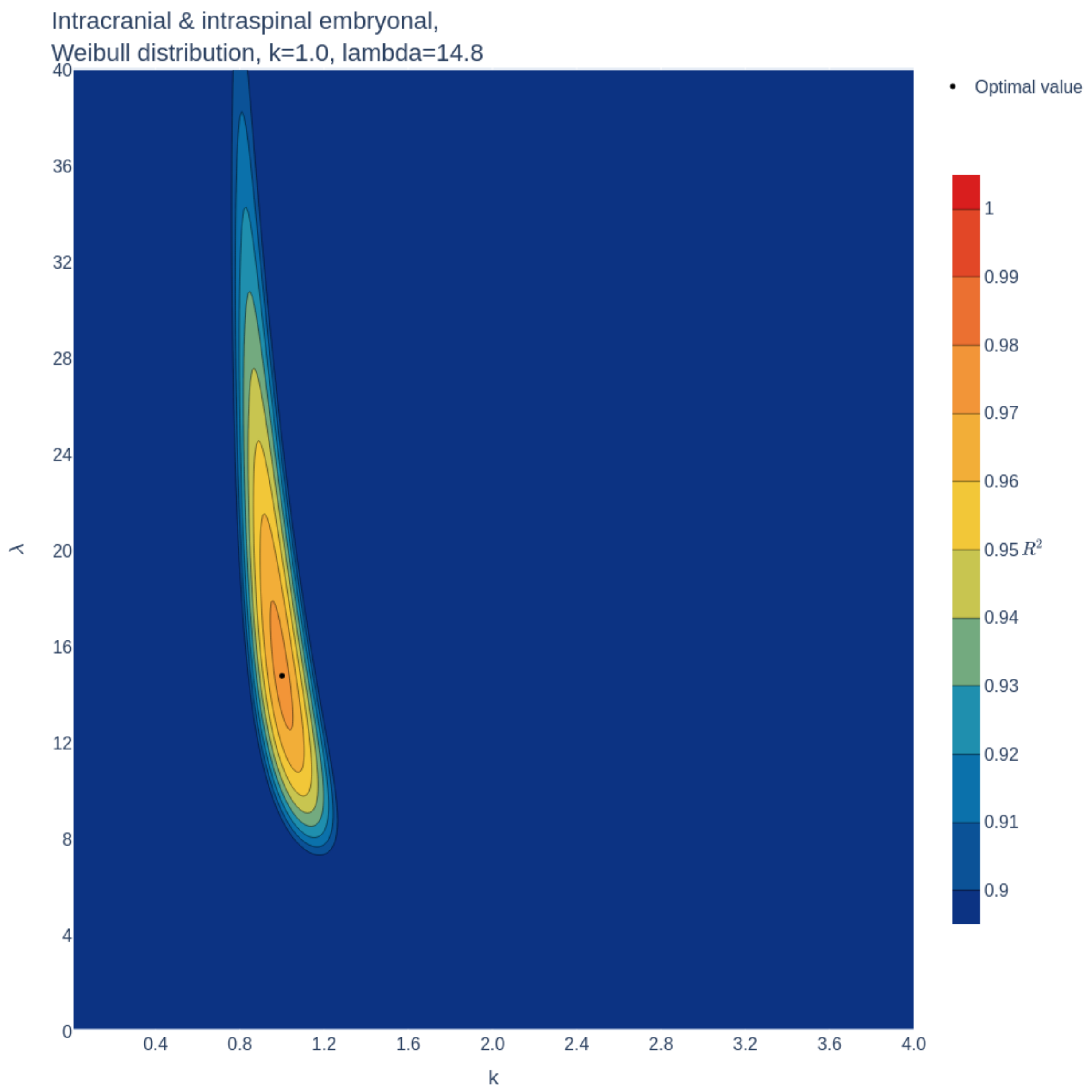

Web Figure 45. Goodness of fit of the Weibull distribution to the age distribution of incidence of intracranial and intraspinal embryonal tumors as a function of various parameter combinations.

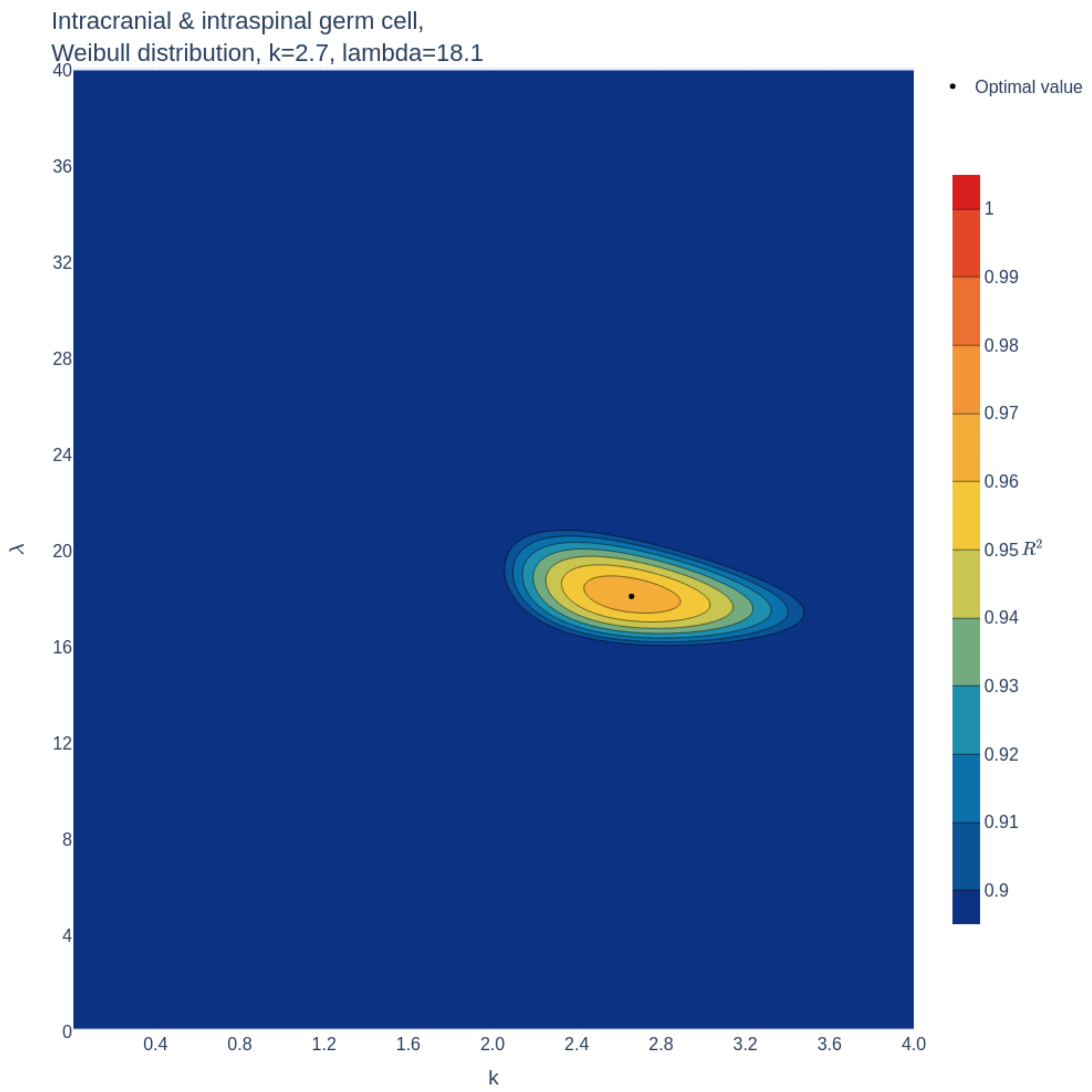

Web Figure 46. Goodness of fit of the Weibull distribution to the age distribution of incidence of intracranial and intraspinal germ cell tumors as a function of various parameter combinations.

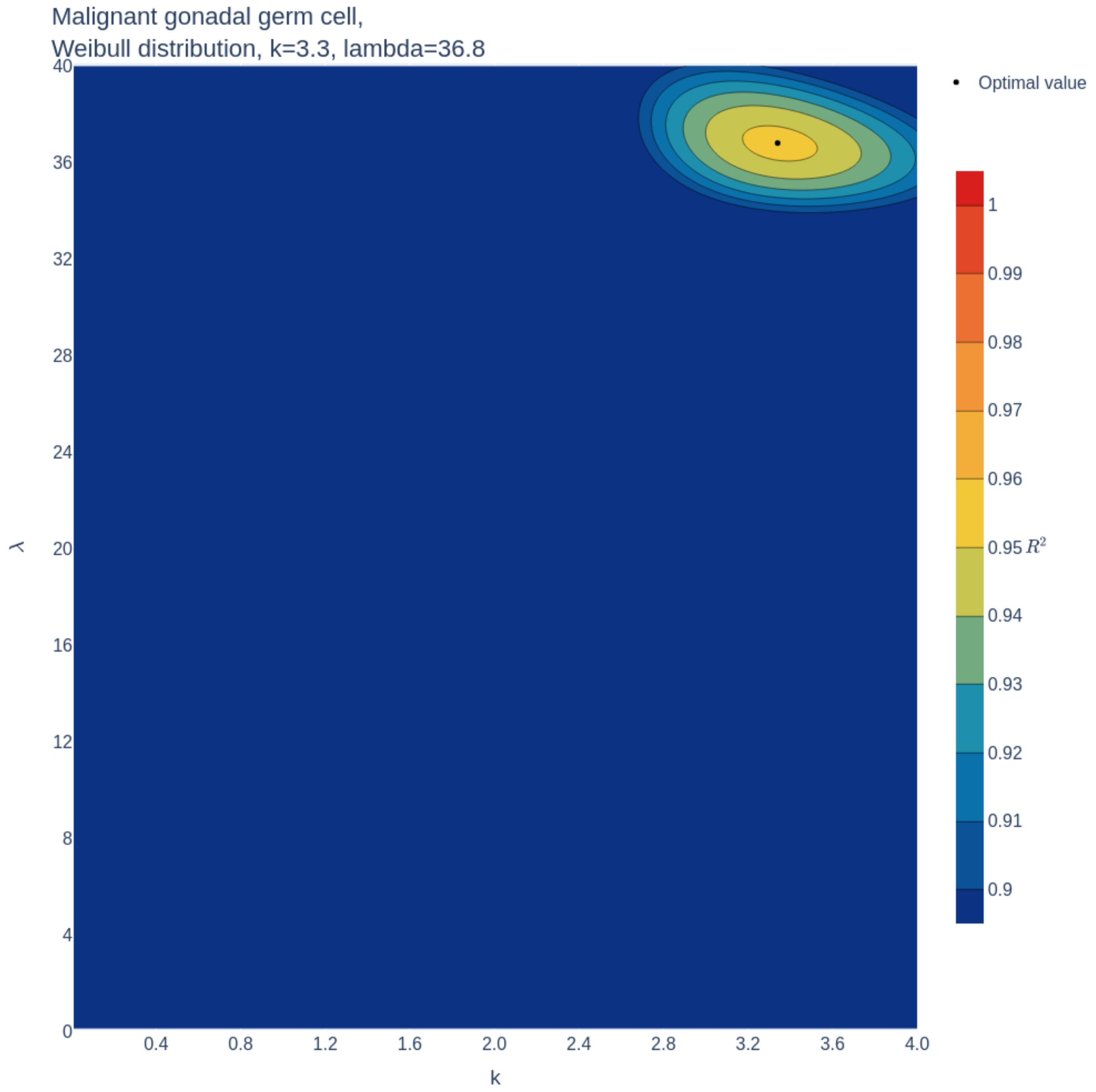

Web Figure 47. Goodness of fit of the Weibull distribution to the age distribution of incidence of malignant gonadal germ cell tumors as a function of various parameter combinations.

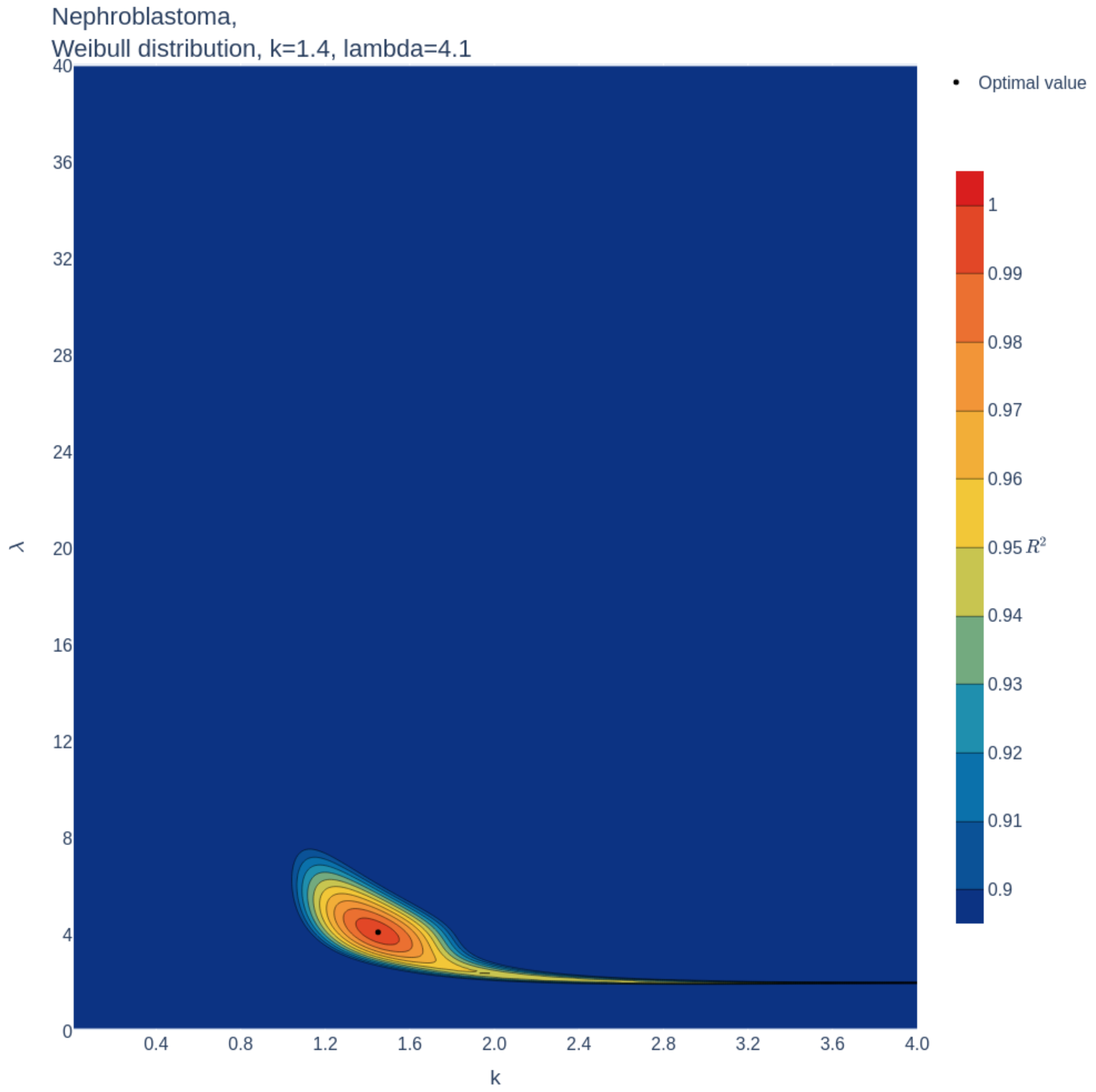

Web Figure 48. Goodness of fit of the Weibull distribution to the age distribution of incidence of nephroblastoma as a function of various parameter combinations.

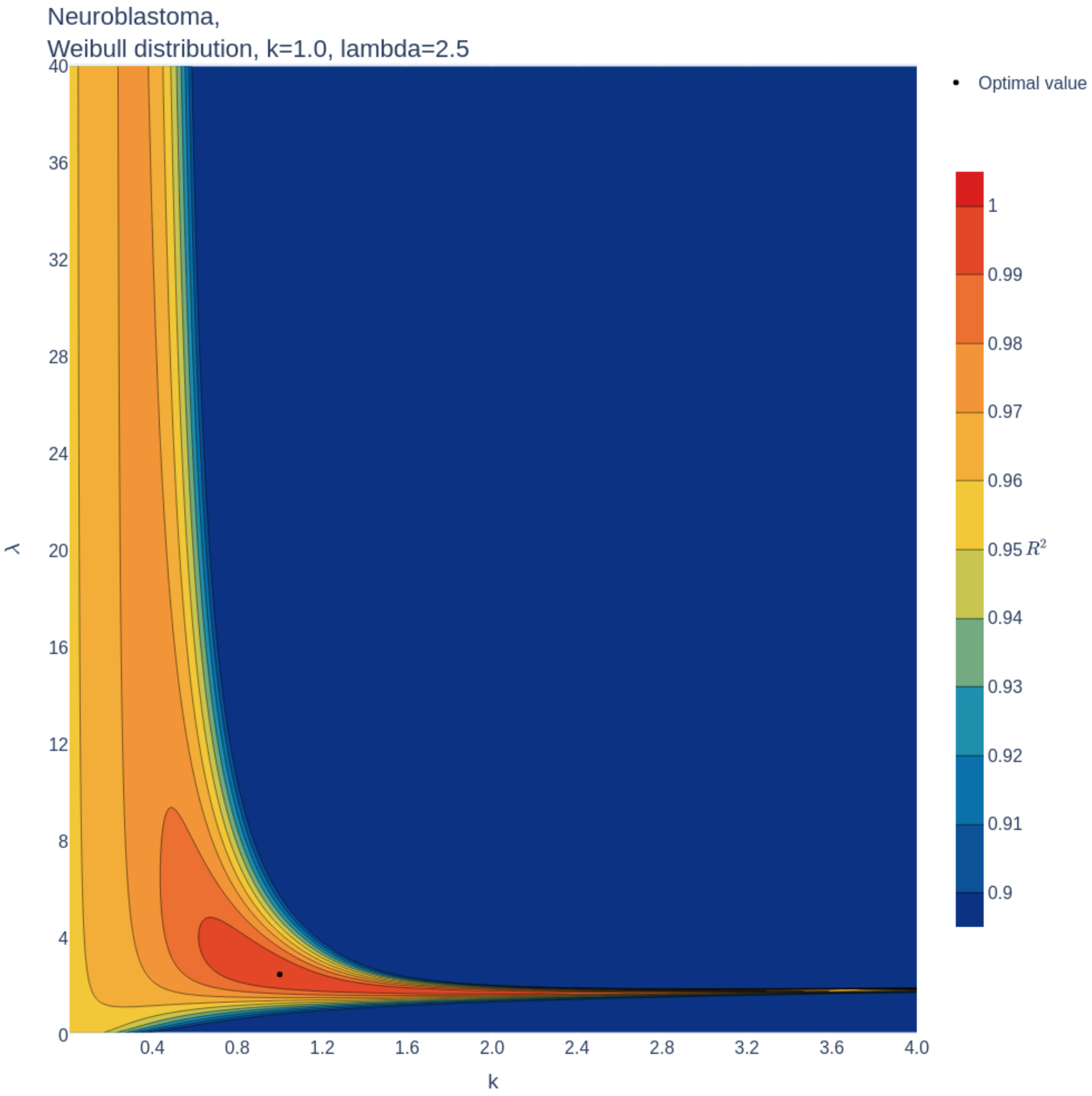

Web Figure 49. Goodness of fit of the Weibull distribution to the age distribution of incidence of neuroblastoma as a function of various parameter combinations.

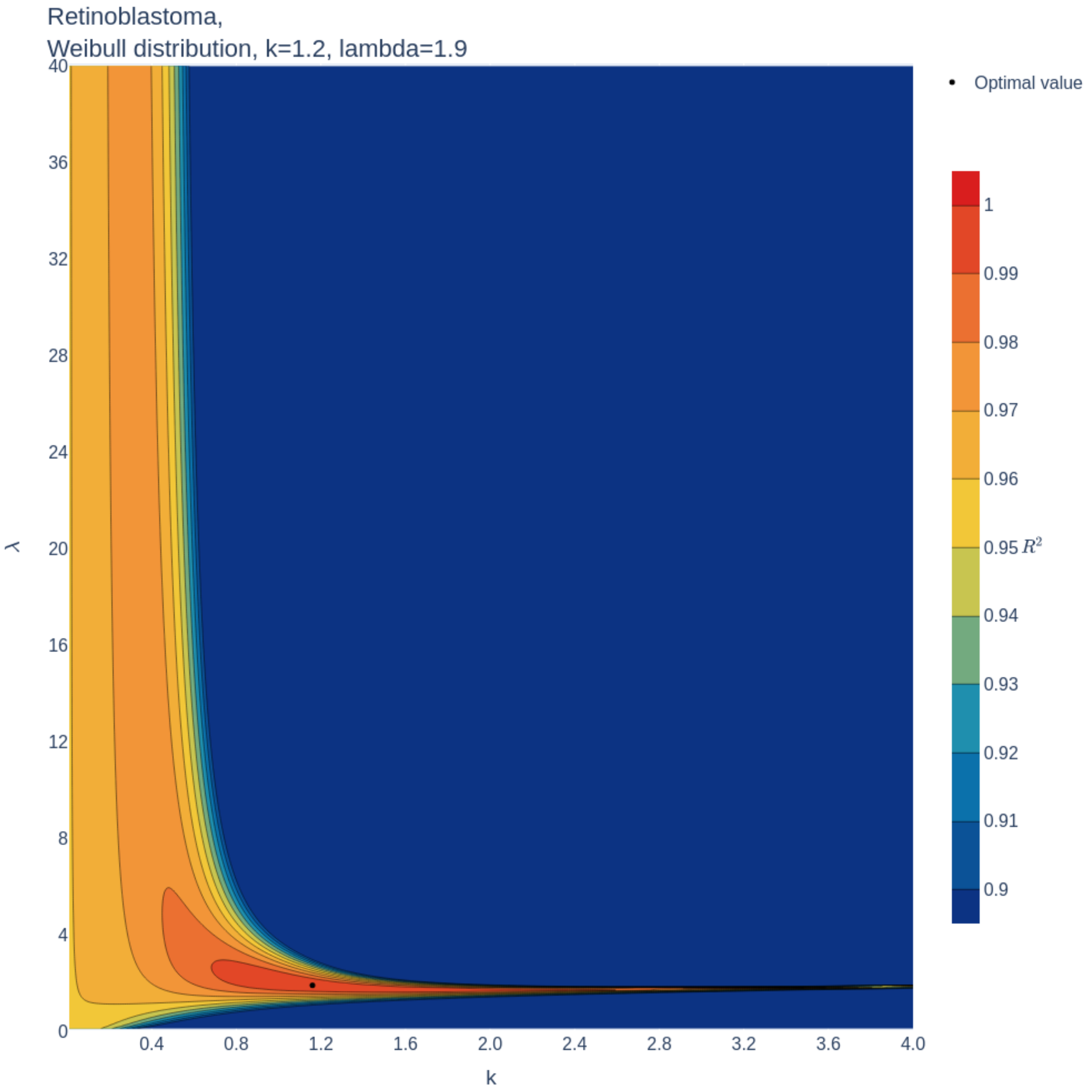

Web Figure 50. Goodness of fit of the Weibull distribution to the age distribution of incidence of retinoblastoma as a function of various parameter combinations.
